# Genome-Wide Identification and Expression Analysis of Adenylate Kinase Family Members in Pepper Under Abiotic Stress

**DOI:** 10.3390/ijms262010213

**Published:** 2025-10-21

**Authors:** Bingxue Han, Kexu Sun, Jingyuan Zhou, Junwei Xu, Aidi Feng, Xiaohong Zhao

**Affiliations:** 1 College of Life Sciences, South China Agricultural University, Guangzhou 510642, China; 18731666313@163.com; 2 College of Agriculture and Biotechnology, Zhejiang University, Hangzhou 310058, China; kexu.23@intl.zju.edu.cn (K.S.); 3230100642@zju.edu.cn (J.Z.); 3230100720@zju.edu.cn (J.X.); adfeng@zju.edu.cn (A.F.); 3 College of Life and Environmental Sciences, Hangzhou Normal University, Hangzhou 311121, China

**Keywords:** pepper, adenylate kinase, abiotic stress, expression profile

## Abstract

Adenylate kinase (ADK), a highly conserved and ubiquitously expressed enzyme in plants, serves as a critical regulator of cellular energy homeostasis and abiotic stress adaptation. While *ADK* families have been characterized in model species (e.g., *Arabidopsis thaliana*, *Oryza sativa*) and crops such as tomato (*Solanum lycopersicum*), the molecular features and stress-responsive roles of *ADK* genes in pepper (*Capsicum annuum* L.) remain uncharacterized. Here, we systematically identified 15 *ADK* genes in pepper (named by chromosomal location) and revealed their evolutionary relationships with orthologs from four plant species, clustering into six conserved groups. The promoters of *CaADKs* were found to contain *cis*-acting elements linked to stress responses, including those responsive to abscisic acid, gibberellin, and low-temperature conditions. Tissue-specific expression profiling highlighted *CaADK9* as a ubiquitously expressed member, suggesting a housekeeping function in basal biological processes. Notably, functional assays under low-temperature and salt stress revealed distinct regulatory patterns: *CaADK11* and *CaADK12* were significantly downregulated, while *CaADK9* was upregulated under salt stress, indicating specialized roles in stress signaling. Additionally, we identified ADK-interacting partners involved in nucleotide homeostasis, providing novel insights into the molecular network underlying pepper’s stress responses. This study represents the first comprehensive analysis of the *CaADK* family, laying a foundation for unraveling ADK-mediated stress adaptation mechanisms in Solanaceous crops.

## 1. Introduction

Adenylate metabolism serves as a fundamental pillar of primary metabolism in living organisms. At its core lies the adenylate cycle, where the interconversion of ATP, ADP, and AMP acts as the “energy currency” driving cellular proliferation and developmental transitions in plants [1]. Adenylate kinase (ADK), a ubiquitously distributed and evolutionarily conserved enzyme, plays a pivotal role in this cycle. It catalyzes the reversible reaction of phosphate transfer between ATP and AMP (ATP + AMP ⇋ 2ADP), a process critical for maintaining adenylate homeostasis within cells [2,3]. Early investigations revealed that ADK also exerts control over the efficiency of energy transfer from mitochondria to hexokinase, laying essential groundwork for understanding its involvement in cellular energy balance regulation [4]. Given the centrality of energy homeostasis in enabling plants to cope with abiotic stresses, ADK is hypothesized to hold vital importance in mediating the stress response of pepper [4].

The “energy charge ratio”, determined by the relative concentrations of AMP, ADP, and ATP, stands as a key regulator of carbohydrate metabolism. This ratio further exerts a profound influence on plant growth, development, and their ability to adapt to external environmental challenges [1,5,6]. Structurally, ADK proteins typically feature three conserved domains: an AMP-binding domain, an ATP-binding domain, and a large central CORE domain [7,8].

Extensive functional studies conducted on model plants and major crops have underscored the biological significance of *ADK*. It is highly conserved across organisms, and its activity has been functionally characterized in model plants (*Arabidopsis thaliana*, *Oryza sativa*) with subcellular localization observed in the cytoplasm, mitochondria, nucleus, and plastids [9,10,11]. In *Arabidopsis thaliana*, disruption of the *ADK* gene *At5g47840* leads to a loss of chloroplast integrity, manifesting as a bleached phenotype during the critical transition from early embryo to seedling development [5]. Another study revealed that disruption of the *ADK* gene *At2g37250* via T-DNA insertion exhibited elevated amino acid accumulation and promoted root elongation [12]. In *Solanum tuberosum* (potato), the downregulation of *StADK* expression results in alterations to adenylate levels and starch content [6]. Beyond regulating developmental processes, *ADK* orchestrates abiotic stress adaptation through dynamic adjustments of adenylate pools, which directly influence stress-responsive gene expression [13]. In *Glycine max*, the expression of *ADK* genes was higher in salt-tolerant varieties than in salt-sensitive ones after salt stress, suggesting that *GmADK* is involved in the salt response [14]. In *Solanum lycopersicum* (tomato), microarray analysis revealed that the *ADK* homolog (SGN-U214214) was downregulated under salt stress, while the *ADK* gene (SGN-U232826) in drought-tolerant species showed significant upregulation under drought conditions [15,16]. Collectively, these studies highlight the conserved yet diverse roles of *ADK* in plant growth, development, and stress resistance, providing a compelling rationale for exploring its function in other crop species, including pepper.

Pepper (*Capsicum annuum* L.), a globally significant vegetable and spice crop, plays a pivotal role in agricultural production, particularly in China. It is rich in capsaicin, carotenoids, and vitamin C and is widely used in food and pharmaceutical industries [17,18]. However, abiotic stresses such as salt and low-temperature stress pose severe threats to pepper metabolism, ultimately leading to reduced yield and quality [19]. Despite the well-documented roles of *ADK* in stress adaptation across other plant species, the molecular characteristics and stress-responsive functions of *ADK* genes in pepper remain largely uncharacterized. This knowledge gap impedes our understanding of how pepper regulates energy homeostasis under stress and restricts the development of stress-tolerant pepper varieties to some extent.

To address these critical knowledge gaps, this study aims to systematically identify and characterize the *ADK* gene family in pepper. We hypothesize that *CaADK* genes in pepper have undergone lineage-specific diversification, with distinct members integrating energy metabolism and hormone-mediated signaling to regulate adaptations to salt, cold, and combined stresses. Their functional divergence is shaped by *cis*-regulatory elements and phylogenetic relationships within Solanaceae. Specifically, we will identify pepper *ADK* genes, analyze their evolutionary relationships, structural properties, and tissue-specific expression patterns, and investigate their transcriptional dynamics under low-temperature and salt stress to unravel their roles in abiotic stress adaptation. By achieving these aims, we hope to deliver the first full-scale analysis of the *CaADK* family in pepper. This work will lay a solid foundation for understanding ADK-mediated stress adaptation in Solanaceous crops, facilitate the development of stress-resistant pepper varieties, and deepen insights into plant stress physiology.

## 2. Results

### 2.1. Genome-Wide Identification and Characterization of CaADK Family Members

Adenylate kinases (ADKs) are evolutionarily conserved enzymes critical for abiotic stress adaptation, including cold, salinity, and drought responses. To identify potential *ADK* homologs in *Capsicum annuum*, we performed a BLAST search against the pepper genome using ADK protein sequences from *Arabidopsis thaliana* as queries, followed by validation of the conserved ADK domain (PF00406). A total of 15 *ADK* genes (annotated as *CaADK*) were identified throughout the *Capsicum annuum* (pepper) genome. These genes were distributed on eight chromosomes and named *CaADK1* to *CaADK15* based on their chromosomal positions (Table 1).

To comprehensively understand the basic characteristics of *CaADK* gene family members, we analyzed their genomic locations, the size of their proteins, instability index (II), grand average of hydropathicity (GRAVY), molecular weights (MW), and isoelectric points (pI). The protein lengths of CaADKs ranged from 112 amino acids (CaADK14) to 536 amino acids (CaADK12), the GRAVY values ranged from −0.466 (CaADK7) to 0.302 (CaADK14), and molecular masses ranged from 12,137.1 Da (CaADK14) to 59,842.7 Da (CaADK12). The pI spanned from 4.67 (CaADK14) to 8.13 (CaADK13). Subcellular localization predictions revealed compartment-specific distributions: six cytoplasmic-targeted, three chloroplast, two mitochondrial, three nuclear, and one cytoskeletal. These findings highlight structural and functional diversification within the *CaADK* family.

### 2.2. Phylogenetic Analysis and Classification of CaADKs

**Figure 1:**
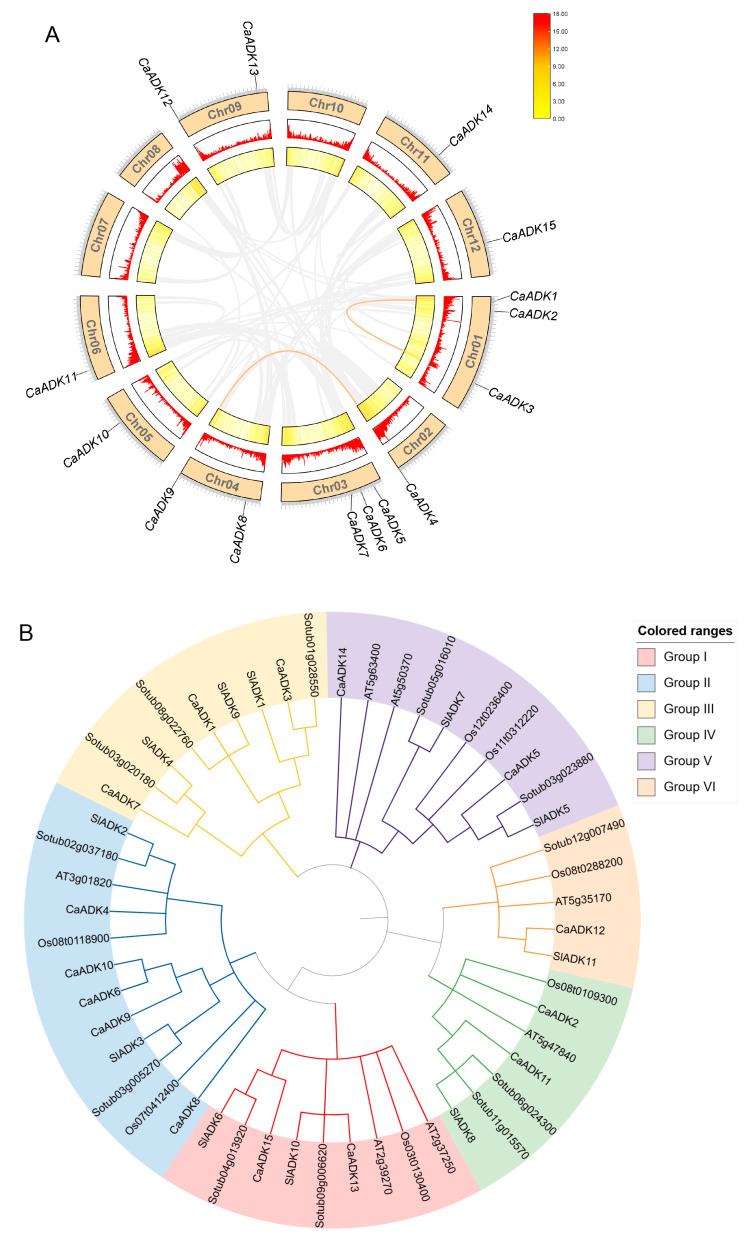
(**A**) *CaADK* gene localization and replication on pepper chromosomes. The outermost orange blocks are representative of the 12 chromosomes, while the middle and inner regions show the density of each chromosome. The curves of orange link the *ADK* genes in pepper. (**B**) Phylogenetic tree of *ADK* in pepper, *Arabidopsis*, rice, potato, and tomato. Different colors correspond to different groups.

To investigate evolutionary relationships within the *ADK* family, we mapped the chromosomal locations of *CaADK* genes and identified 15 members localized across nine chromosomes (Figure 1A). Chromosomes 01 and 03 had the greatest number of predicted *CaADKs*, with three genes each, and no *CaADK* existed on chromosomes 07, 08, and 10. Two genes were present on chromosomes 04 and 09, respectively, and only one *CaADK* was found on each of the other five chromosomes.

A maximum-likelihood phylogenetic tree was constructed using full-length ADK protein sequences from *Capsicum annuum* (15), *Arabidopsis thaliana* (7), *Oryza sativa* (7), *Solanum lycopersicum* (11), and *Solanum tuberosum* (12). The phylogenetic tree resolved *ADKs* into six distinct clades (Group I–Group VI), with *CaADK* genes represented in all clades (Figure 1B). Group II was the largest, containing 12 ADKs: 5 from pepper, 1 from *Arabidopsis*, 2 from rice, and 2 each from tomato and potato, suggesting conserved roles in basal metabolism. Clade V, the second-largest clade, included 10 ADKs: 2 of each of the five species. Clades I and III each contained 9 ADKs, Group IV had 7 members, and Group VI had the fewest, with only 5 members from pepper, *Arabidopsis thaliana*, rice, potato, and tomato. These phylogenetic relationships indicate that *CaADKs* share evolutionary conservation with orthologs in other plants, particularly Solanaceous species, while also exhibiting lineage-specific diversification.

**Figure 2:**
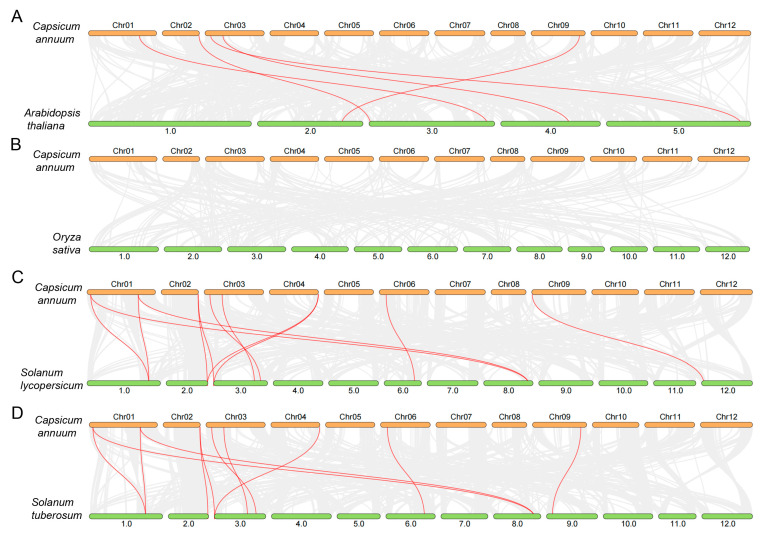
Collinearity analysis of the *ADK* family genes between pepper and (**A**) *Arabidopsis* thaliana; (**B**) rice; (**C**) tomato; (**D**) potato. Red lines indicate homologous genes.

Gene duplication, including tandem and segmental duplication events, serves as a key mechanism driving functional innovation and adaptive evolution in plants. Our analysis revealed two segmental duplication events in the pepper genome: *CaADK1*/*CaADK3* and *CaADK4*/*CaADK9* (Figure 1A). Additionally, homology analyses were performed among pepper, *Arabidopsis*, rice, tomato, and potato *ADK* genes. These analysis results identified 11 orthologous *ADK* pairs between pepper and potato (Figure 2D), 11 with tomato (Figure 2C), and 5 with *Arabidopsis* (Figure 2A), while no orthologs were detected in rice, reflecting monocot-dicot divergence (Figure 2B). Studies on the evolution of Solanaceous plants have revealed their genome evolution and polyploidization events, which may be important forces shaping the evolution of the *CaADK* gene family [20].

### 2.3. Diversity Among CaADK Family Members

**Figure 3:**
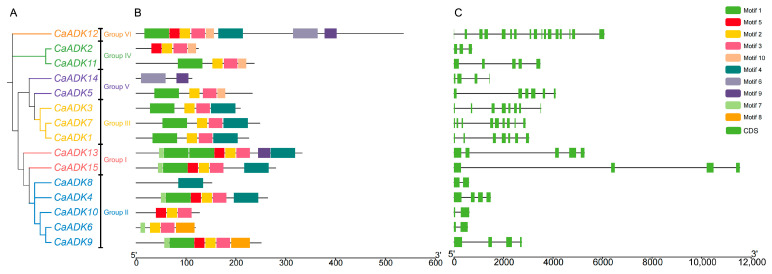
Gene structure and characterization of conserved motifs of *CaADKs* from pepper. (**A**) Maximum likelihood phylogenetic tree of *CaADKs*. (**B**) The motifs of CaADK protein. (**C**) Gene structure of *CaADKs*. Green boxes represent exons, and lines represent introns.

To unravel the structural divergence of *CaADK* family members, we systematically analyzed their genetic architectures. A maximum likelihood phylogenetic tree constructed with MEGA X is shown in Figure 3A, which is consistent with the result in Figure 1B. To further explore conservation patterns within the family, we analyzed conserved motifs and exon-intron organizations in the context of this phylogenetic tree. Ten motifs were identified in CaADK proteins and annotated as Motifs 1 to 10 (Figure 3B and Table 2). Conserved motif analysis of CaADK proteins revealed that Motif 2 and Motif 3 were ubiquitously conserved, present in 13 of the 15 genes. Motif 1 was also highly conserved, existing in 10 genes (67%). In contrast, Motif 6 was restricted to CaADK12 and CaADK14, while Motif 8 was lineage-specific—found only in CaADK6 and CaADK9 of Group II—suggesting it may contribute to clade-specific regulatory functions. Notably, within Group II, CaADK8 contained 1 motif, differing from other members in the clade; such divergence may result from functional specialization or genome annotation inaccuracies, potentially leading to distinct expression profiles and functional modifications among family members.

Beyond motif conservation, structural divergence was evident in exon-intron organization. The number of exons in the *CaADK* family ranged from 2 to 17: *CaADK12* had the maximum number of exons, while *CaADK8*, *CaADK10*, and *CaADK6* (all in Group II) had the shortest gene length with 2 exons. We observed that exon lengths within each clade were highly conserved, whereas introns exhibited substantial size variation, reflecting selection for conserved coding sequences and flexible regulatory evolution through intronic plasticity (Figure 3C). For example, in Group I, *CaADK13* and *CaADK15* had long gene lengths due to considerably extended introns, despite containing only 5 and 4 exons, respectively, which may enable fine-tuning of their expression through intronic regulatory elements.

### 2.4. GO Analysis of ADKs in Pepper

**Figure 4:**
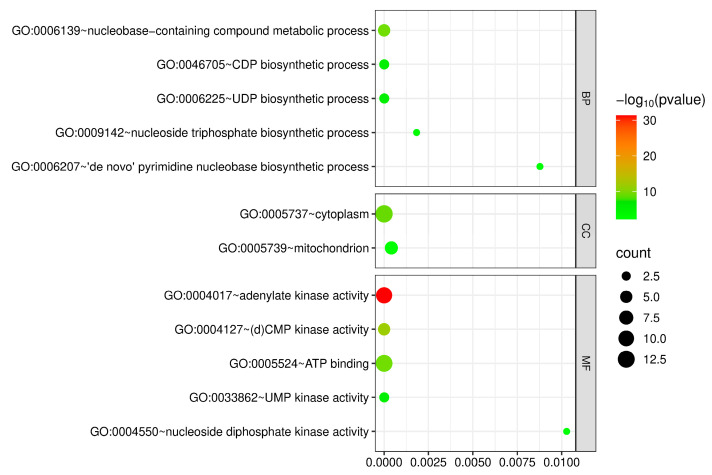
The gene ontology (GO) annotations assigned to *ADKs* in pepper. GO terms were classified into three main categories: “BP” for biological process, “CC” for cellular component, and “MF” for molecular function. The size of the dot bubble represents the number, and the color represents the *p*-value of genes for that GO term.

Gene ontology (GO) enrichment analysis of *CaADKs* was conducted, categorizing terms into biological process (BP), cellular component (CC), and molecular function (MF) (Figure 4). In the BP category, *CaADKs* were enriched in nucleotide metabolism-related processes, including nucleobase-containing compound metabolic process (GO:0006139), CDP biosynthetic process (GO:0046705), UDP biosynthetic process (GO:0006225), de novo’ pyrimidine nucleobase biosynthetic process (GO:0006207), and nucleoside triphosphate biosynthetic process (GO:0009142). For CC terms, the most prominent categories were cytoplasm (GO:0005737) and mitochondrion (GO:0005739), which were consistent with the subcellular localization prediction results. The MF category exhibited the strongest enrichment, with *CaADKs* significantly associated with adenylate kinase activity (GO:0004017). Collectively, these results indicate that *CaADKs* primarily function in nucleotide metabolism and adenylate kinase activity, with their subcellular localization supporting roles in cellular energy homeostasis.

### 2.5. Protein Secondary and Tertiary Structure Analysis of CaADKs

**Figure 5:**
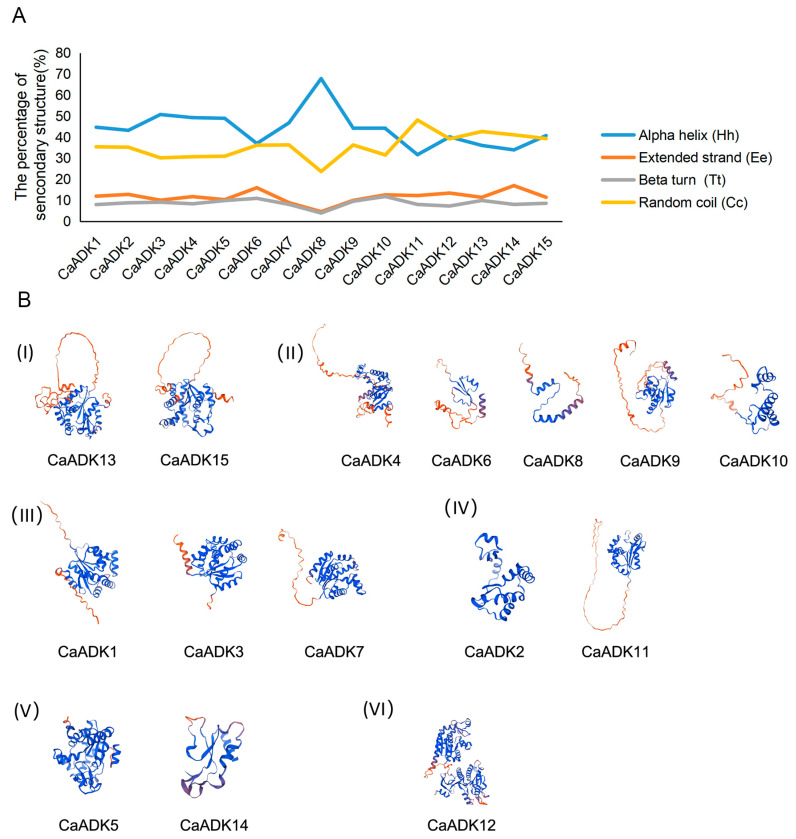
(**A**) The secondary structure analysis of ADKs in pepper. (**B**) Tertiary structure analysis of ADKs in pepper. The color of the bands varies from red to blue, indicating that the protein structure is from the N-terminus to the C-terminus.

To explore structural conservation and diversification of ADK proteins, we performed comparative analysis focusing on pepper CaADKs, which helps elucidate conserved catalytic domains and lineage-specific adaptations underlying functional divergence across plant species. Secondary structure analysis of ADK proteins in pepper revealed four elements: alpha helix (Hh), extended strand (Ee), beta turn (Tt), and random coil (Cc) components (Figure 5A). Alpha helixes and random coils dominated CaADK secondary structures, whereas beta turns were minimal, suggesting limited rigid loops. Such variations in secondary structure—particularly in the proportion of alpha helices (critical for catalytic domain stability) and random coils (involved in flexible protein interactions)—may contribute to functional divergence, such as differences in allosteric regulation among stress-responsive isoforms.

Building on the secondary structure analysis, we further predicted the tertiary structures of CaADK proteins through homology modeling, which results from additional coiling and folding of secondary structural elements. SWISS-MODEL analysis revealed that members of the same phylogenetic subgroup shared high similarity in tertiary structure (Figure 5B), aligning with their conserved evolutionary origin and suggesting functional redundancy within subgroups. Visualizing these tertiary conformations provides mechanistic insights into how structural differences may drive functional divergence among distinct CaADK members.

### 2.6. Cis-Acting Element Analysis of CaADKs

To investigate the regulatory mechanisms of the ADK genes, we analyzed *cis*-acting elements in the 2-kb promoter regions upstream of CaADK coding sequences using PlantCARE. These elements included those associated with stress responses (e.g., low-temperature responsiveness, drought-inducibility) and phytohormone signaling (e.g., abscisic acid responsiveness, gibberellin responsive), with detailed information provided in Table S5.

**Figure 6:**
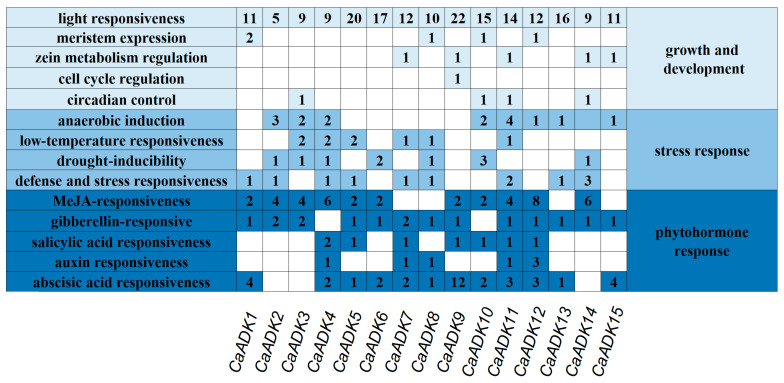
Analysis of *cis* elements in the promoter of *ADK* genes in pepper. Different shades of blue indicate the presence of *cis* elements involved in different biological processes, and the number in each cell indicates the number of *cis*-acting elements in each gene. White cells indicate the absence of the *cis*-acting element. Different shades of blue on both sides of the square correspond to distinct biological processes.

We found variable numbers of *cis* element across *CaADK* promoters, with stress- and hormone-related elements being most abundant (Figure 6). Specifically, stress-responsive elements included those for anaerobic induction, low-temperature responsiveness, drought-inducibility, and defense/stress responsiveness; phytohormone-responsive elements such as abscisic acid responsiveness, MeJA responsiveness, gibberellin responsive, and salicylic acid responsiveness. Notably, all *CaADK* promoters contained light-response elements, implicating their potential role in coordinating with light-dependent processes. Approximately 87% (13 of 15) of these genes harbored gibberellin responsive elements, 80% (12 of 15) contained abscisic acid responsiveness elements, and 73% (11 of 15) had MeJA-responsiveness elements. The number and type of *cis* elements differed among the promoters of each *CaADK* gene, reflecting their distinct functional requirements. Collectively, these data—particularly the enrichment of stress- and hormone-related elements—support that *CaADK* genes are integrated into abiotic stress regulatory networks, with their unique *cis* element profiles enabling specific responses to diverse environmental cues.

### 2.7. Tissue-Specific Expression Patterns of CaADKs

**Figure 7:**
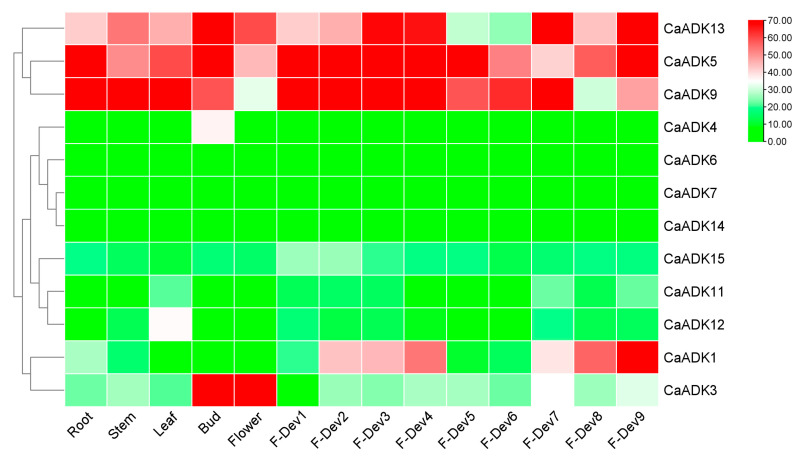
Expression levels of *ADK* genes in different organs of pepper, with deeper hues of red indicating higher expression and cooler tones suggesting lower expression levels. F-Dev1 to F-Dev4 denote fruit from 0 to 5 cm; F-Dev5 denotes ripe green fruit; F-Dev6 denotes fruit at the color change stage; and F-Dev7 to F-Dev9 denote fruit at 3, 5, and 7 d after color change, respectively.

Tissue-specific expression profiles of 15 *CaADKs* (analyzed using transcriptome data from ‘Zunla-1’) revealed distinct transcriptional patterns across five vegetative tissues and nine fruit developmental stages (Figure 7, Table S2). Six *CaADK* genes (*CaADK4*, *CaADK7*, *CaADK14*, *CaADK15*, *CaADK11*, and *CaADK12*) showed minimal expression across all tissues, suggesting they may be functionally specialized or induced only under specific conditions, whereas *CaADK6* was silenced in different parts of pepper and fruit growth stages. In contrast, *CaADK13*, *CaADK5*, and *CaADK9* were highly expressed in multiple tissues, including roots, stems, leaves, and fruits, indicating potential housekeeping roles in basal biological processes. Notably, *CaADK3* exhibited preferential expression in floral tissues, implying a role in reproductive development. *CaADK1* showed peak expression at 7 days post-color transition in fruits, suggesting involvement in fruit ripening or pigment accumulation. These tissue-specific patterns highlight functional diversification within the *CaADK* family, with some members contributing to general cellular processes and others to tissue-specific development.

### 2.8. Transcriptome Analysis of CaADKs in Response to Abiotic Stresses and Exogenous Hormone Treatments

**Figure 8:**
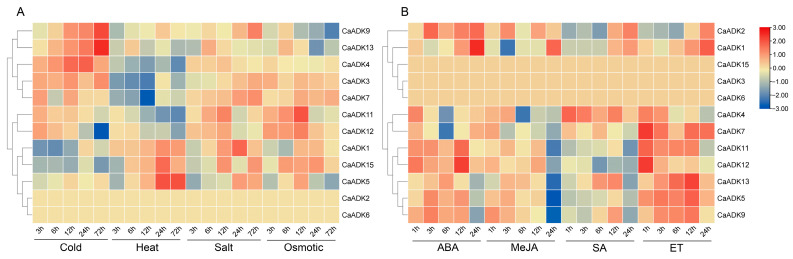
Expression analysis of *CaADK* genes under abiotic stresses and exogenous hormone treatments. (**A**) The expression of *CaADKs* after 3, 6, 12, 24, and 72 h of cold, heat, salt and osmotic stresses. (**B**) The expression of *CaADKs* after 1, 3, 6, 12, and 24 h of ABA, MeJA, SA, and ET stresses.

To elucidate the functional roles of the *ADK* gene family in stress adaptation, we profiled *CaADK* expression under abiotic stress and exogenous hormone treatments. The expression dynamics of these genes were consistent with the timing of their upregulation or downregulation in the transcriptome (Figure 8).

The expression levels of *CaADK* genes varied under different treatments (Figure 8A). Several genes exhibited significant and consistent expression trends. Under low-temperature treatment, the expression levels of *CaADK9* and *CaADK13* were upregulated, with the highest expression observed after 72 h of treatment—this sustained upregulation suggests that these two members may contribute to long-term cold acclimation. *CaADK5* expression was upregulated under both heat and salt stresses, while *CaADK7* was specifically upregulated under salt stress, indicating potential roles in mediating responses to multiple or specific stressors. In contrast, *CaADK11* and *CaADK12* were suppressed under both cold and heat stress, implying they may be involved in negative regulation or energy conservation under these conditions. These findings demonstrate that *CaADKs* are critical regulators of abiotic stress adaptation, particularly in low-temperature and salinity stresses.

Exogenous hormone treatments revealed that most *CaADKs* experienced transient suppression (1–6 hpt) followed by gradual recovery (6–24 hpt) (Figure 8B), suggesting a conserved early response mechanism to hormonal cues. Conversely, *CaADK1* was significantly upregulated at 12 h under salicylate treatment, while *CaADK9* was suppressed under MeJA treatment, indicating member-specific interactions with hormone signaling pathways. Notably, *CaADK2* and *CaADK6* were transcriptionally silent under abiotic stresses, and *CaADK3*/*6*/*15* remained unresponsive to hormonal treatments, suggesting that they are insensitive to changes in the external environment. These distinct expression patterns under stress and hormone treatments indicate that the *CaADK* family employs a specialized regulatory network to mediate pepper’s adaptation to environmental challenges, with specific members likely acting as key nodes in stress signaling pathways.

### 2.9. Expression Patterns of CaADKs Under Abiotic Stress

**Figure 9:**
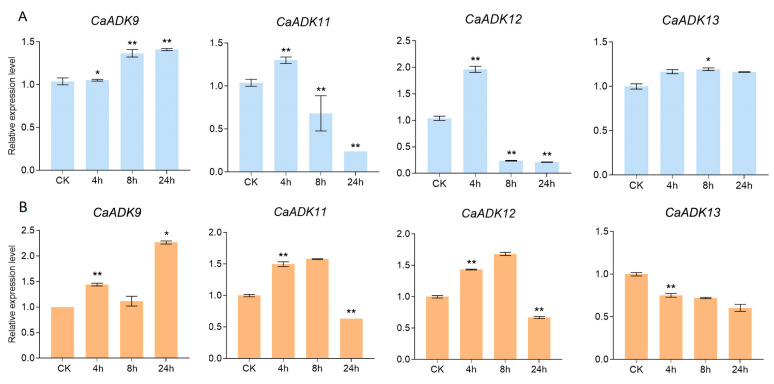
Expression analysis of *CaADK* under low-temperature stress and salt stress at different time treatments was analyzed using RT-qPCR: (**A**) Expression analysis of *CaADK* under low-temperature stress. (**B**) Expression analysis of *CaADK* under salt stress. Data were shown as means; errors are shown as ±SD (*t*-test, * *p* < 0.05, ** *p* < 0.01).

To validate the expression dynamics of *CaADK* genes under abiotic stress, four candidate genes (*CaADK9*, *CaADK11*, *CaADK12*, and *CaADK13*) in pepper—selected based on their differential expression in transcriptome data—were analyzed using RT-qPCR analysis at 0 h, 4 h, 8 h, and 24 h post treatment (hpt) under both low-temperature and salt stress (Figure 9).

Under low-temperature stress, *CaADK11* and *CaADK12* were significantly downregulated by over 4-fold after 24 h of low-temperature stress (*p* < 0.01, *t*-test), consistent with transcriptome results. *CaADK9* showed upregulation at 8 hpt, while *CaADK13* exhibited a slight upregulation throughout the treatment period (Figure 9A). Under salt stress, *CaADK11*, *CaADK12*, and *CaADK13* were significantly downregulated at 24 hpt, whereas *CaADK9* was markedly upregulated at all time points (Figure 9B). Notably, *CaADK11* and *CaADK12* showed a transient upregulation at 4 h under salt stress, indicating they may participate in early stress sensing, while *CaADK9* maintained elevated expression, suggesting a role in sustained salt stress response. These results confirm that *CaADK* genes exhibit dynamic and divergent expression patterns in response to low-temperature and salt stress, with functional specialization likely enabling fine-tuning of pepper’s stress adaptation mechanisms.

### 2.10. PPI Network Prediction Analysis of ADK Genes

**Figure 10:**
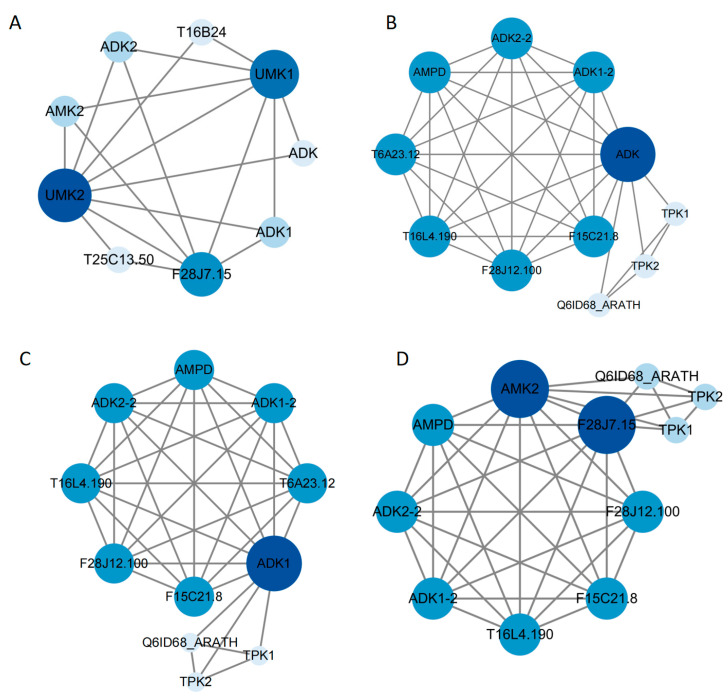
Protein–protein interaction (PPI) networks for ADK genes in *Arabidopsis thaliana*: (**A**) Predictive analysis of ADK interaction networks in *Arabidopsis*. (**B**) At2g37250 (ADK, CaADK13, 15 homologous gene). (**C**) At5g63400 (ADK1, CaADK5 homologous gene). (**D**) At5g47840 (AMK2, CaADK2, 11 homologous gene) and At3g01820 (F28J7.15, CaADK4, 6, 9, and 10 homologous genes). At the network nodes, the circles represent proteins, dark blue circles represent homologous genes and proteins associated with abiotic stress, light blue circles represent other interacting proteins, and connecting lines indicate associations between proteins. The analysis required a minimum engagement score of 0.150, with other parameters set to default values.

Proteins play crucial roles in mediating molecular interactions and cellular signaling pathways. To explore the molecular networks underlying ADK-mediated stress responses, we predicted protein-protein interaction (PPI) networks using *Arabidopsis* homologs of CaADKs (leveraging *Arabidopsis*’ well-characterized PPI data) and inferred potential interactions of pepper ADKs based on these orthologous relationships, aiming to identify functional partners in nucleotide metabolism and stress signaling (Figure 10A). The results showed that four *Arabidopsis* homologs of CaADKs interacted with conserved protein partners: At2g37250 (homologs of CaADK13 and CaADK15, Figure 10B), At5g63400 (homolog of CaADK5, Figure 10C), At5g47840 (homologs of CaADK2 and CaADK11, Figure 10D), and At3g01820 (homologs of CaADK4, CaADK6, CaADK9, and CaADK10; Figure 10D). These homologs interacted with core proteins, including ADK1-1, ADK1-2, AMPD (adenylate deaminase), F15C21.48, F28J12.100, and T16L4.190, with low-degree interactions with TPK1, TPK2, and Q6ID68_ARATH. Notably, interacting proteins such as AMPD (involved in adenylate catabolism) and ADK1 homologs may regulate adenylate kinase activity by modulating nucleotide pool balance. Consistent with this, previous studies reported that ADK1 elevates intracellular ATP levels to fuel glycolysis and oxidative phosphorylation [21], supporting a conserved role of these interactions in energy metabolism. Collectively, these predictions suggest that CaADKs coordinate metabolic adaptation to stress through a conserved network of interactions with nucleotide homeostasis-related proteins, highlighting their role in integrating energy metabolism and stress signaling.

## 3. Discussion

Adenylate kinase (ADK) is a ubiquitous enzyme that mediates the reaction generating ADP (ATP + AMP ⇋ 2ADP), thereby maintaining cellular energy homeostasis. Beyond its role in energy balance, ADK modulates plant growth, developmental transitions, and adaptation to abiotic stresses [12,22]. However, the *ADK* gene family in pepper (*Capsicum annuum* L.) has not been thoroughly studied. In this study, we identified 15 *CaADK* genes in the pepper genome, a number comparable to ADK families in other crops—13 in rice [23], 11 in tomato [24], and 11 in alfalfa [25]—suggesting conserved expansion patterns of the *ADK* family across angiosperms. We characterized their physicochemical properties, phylogenetic relationships, structural features, GO enrichment, protein structures, *cis* elements, protein interactions, and expression patterns under abiotic stresses. These results enhance our understanding of *CaADK* genes and their roles in plant responses to abiotic stresses.

Phylogenetic analysis classified CaADKs from five plant species into six clades, with genes within the same clade sharing closer evolutionary relationships. This clustering—CaADK13/15 in Clade I, CaADK4/6/8/9/10 in Group II, and so on—revealed conserved functional architectures, as evidenced by clade-specific motif distributions and exon-intron organizations. CaADK1, 3, and 7 in Clade III shared homology with UMP-CMP kinases and exhibited strong sequence homology to their *Arabidopsis* orthologs. The 10 identified conserved motifs, particularly their consistent arrangement within clades, suggest potential functional redundancy among closely related members. For example, in Clade III, CaADK1, 3, and 7, which share homology with UMP-CMP kinases and exhibit strong sequence homology to their *Arabidopsis* orthologs, may have overlapping functions in nucleotide metabolism. On the other hand, lineage-specific motifs (e.g., Motif 8 in Group II) may drive functional specialization [26]. These unique motifs could enable interactions with distinct sets of regulatory or substrate proteins, allowing ADKs to participate in novel stress-responsive pathways in pepper. Exon counts ranging from 2 to 17 further highlight the evolutionary dynamism of the family. Conserved exon lengths suggest that the core catalytic domains of ADKs are under strong purifying selection, ensuring the maintenance of their fundamental enzymatic activity. Notably, homology analyses revealed that pepper *ADKs* share the most orthologous pairs with potato and tomato, underscoring close evolutionary ties among Solanaceous crops [27]. The Solanaceae family has evolved in diverse ecological niches, and the conservation of *ADK* orthologs among its members suggests that these genes have been co-opted to mediate stress responses relevant to the family’s typical habitats, such as arid or saline environments. As depicted in Figure 2, orthologous genes were not identified in rice. The absence of orthologous pairs with rice aligns with prior observations of limited collinearity between monocots and dicots [28], reinforcing the evolutionary divergence of ADK families between these lineages. Collectively, these results underscore the evolutionary divergence of the *ADK* gene family between monocotyledonous and dicotyledonous plant species. Therefore, we can predict the gene functions of pepper based on those of other nightshade plants.

Tissue-specific expression profiling demonstrated that *CaADK* genes exhibited ubiquitous distribution across various tissues, mirroring the tissue-dependent specialization reported for *ADKs* in other plants [28]. *CaADKs* were detected in all examined tissues and throughout fruit development stages in pepper. Several *CaADK* family members, including *CaADK5*, *CaADK9*, and *CaADK13*, were identified as key regulators of pepper growth and development, as evidenced by their widespread expression in both vegetative and reproductive tissues. In plants, energy demands vary across tissues and developmental stages. The high expression of these *CaADKs* in roots, stems, and leaves aligns with roles in carbohydrate metabolism, a critical process for plant growth. Carbohydrates are not only energy sources but also signaling molecules, and *ADKs* may play a dual role in regulating both energy availability and carbohydrate-mediated signaling in these tissues.

In this study, we analyzed 14 *cis*-regulatory elements associated with stress responses and hormone regulation, as detailed in Table S5 and Figure 6. *Cis*-acting element analysis and stress-responsive expression data collectively illuminate the roles of specific *CaADKs* in abiotic stress adaptation. *CaADK9*, whose promoter contains abscisic acid (ABA)-responsive elements, was consistently upregulated under salt stress in both RNA-seq and RT-qPCR analyses. Abscisic acid (ABA) regulates seed germination, development, and stress responses [29]. Previous studies have shown that ABA is a typical plant hormone associated with abiotic stress, promoting the expression of stress-related genes and playing an important role in responses to external stress, such as salt stress [30,31]. In addition, expression analysis could provide insight into gene functions. *ADK* genes responded rapidly to biotic and abiotic stresses such as salt stress [32] and also affect ATP metabolism and adenylate balance. The upregulation of *CaADK9* under salt stress suggests that it may act through ABA-dependent pathways. One possible mechanism is that *CaADK9* regulates ATP regeneration to fuel stress-responsive processes such as ion transport. Under salt stress, plants need to maintain ion homeostasis, which requires energy for the operation of ion transporters.

*CaADK12* emerges as a critical regulator of combined salt and cold stress tolerance. Analysis of *cis*-acting elements within the promoter region of *CaADK12* revealed the presence of MeJA, GA, and SA responsive elements, positioning it at the intersection of multiple hormone-mediated stress response pathways. Gibberellin (GA) regulates stress responses by preventing protein degradation through the activation of DELLA protein, which is a repressor on the GA signaling pathway [33]; salicylic acid in plants mediates cross-talk between stress-signaling pathways and enhances cold tolerance [34,35]; and MeJA drives cold-stress responses in tomato by upregulating abscisic acid (ABA) biosynthesis [36]. Exogenous MeJA mediates the expression of genes across diverse pathways. For example, it impacts processes such as chlorophyll biosynthesis and degradation, antioxidant enzyme systems, and jasmonic acid (JA) biosynthesis, thereby enhancing the plant’s tolerance to abiotic stresses [37]. *CaADK12* was found to possess a substantial number of MeJA-responsive *cis* elements. This discovery strongly implies its potential involvement in the regulatory mechanisms of the MeJA response to stress. Additionally, results from exogenous hormone treatments indicated that the expression level of *CaADK12* was significantly downregulated after MeJA and SA treatments. Transcriptomic and RT-qPCR data confirm downregulation of *CaADK12* under both salt and cold stress, with prolonged suppression suggesting an energy-conserving strategy to prioritize critical stress-acclimation processes. When plants are exposed to multiple stresses, energy becomes a scarce resource, and downregulating certain *ADKs* may redirect energy to more essential processes such as the synthesis of stress-protective proteins or osmolytes. The role of *CaADK12* in this energy reallocation under combined stress conditions highlights the complexity of ADK-mediated stress responses.

Structural analyses further support functional specialization. Secondary structures dominated by alpha helices and random coils provide flexibility for allosteric regulation, which is crucial for enzymes such as ADKs, as it allows them to respond to changes in the cellular environment. While conserved tertiary structures within clades ensure core catalytic functionality, allowing ADKs to perform their basic role in nucleotide phosphorylation-dephosphorylation reactions [21,38]. Subcellular localization predictions indicated that CaADK proteins are localized to mitochondria and chloroplasts. Consistent with our findings, ADKs in plants are predominantly localized in the chloroplast stroma and mitochondrial intermembrane space, where they account for 90% of total enzymatic activity [38,39]. These features, combined with protein-protein interaction predictions linking CaADKs to nucleotide homeostasis proteins (AMPD, ADK1 homologs), reinforce their role as integrators of energy metabolism and stress signaling. For instance, interactions with AMPD (adenosine monophosphate deaminase) could allow CaADKs to sense and respond to changes in AMP levels, which are key indicators of cellular energy status. In times of stress, when AMP levels rise, this interaction could trigger a cascade of events to restore energy homeostasis, either by activating ADK to convert AMP and ATP to ADP or by initiating other metabolic or signaling pathways.

Previous studies demonstrate that the *ADK* gene *At5g47840* is vital for chloroplast integrity and ensures plants grow properly from embryo to seedling development [5]. *CaADK11*, a chloroplast-localized homolog of *Arabidopsis At5g47840*, contains abundant anaerobic induction and low-temperature responsive elements. Both transcriptome and RT-qPCR data collectively confirmed that the expression of *CaADK11* was downregulated under salt and cold stress conditions, implying its role in coordinating chloroplast function under stress. By reducing non-essential metabolic activity in the chloroplast, *CaADK11* may preserve resources to maintain basic chloroplast functions, such as the synthesis of essential proteins and lipids. This aligns with subcellular localization predictions, as chloroplast and mitochondrial ADKs are known to regulate energy balance in organelles critical for stress adaptation. Based on these findings, we propose that the regulation of *CaADK11* in response to stress thus represents a fine-tuned mechanism to balance energy consumption and organelle function under adverse conditions.

In summary, our findings reveal that the *CaADK* family has evolved conserved and lineage-specific features, with distinct members mediating pepper’s response to cold and salt stress through differential expression, structural specialization, and interactions with nucleotide metabolism proteins. These provide a foundational characterization of *CaADKs*.

## 4. Materials and Methods

### 4.1. Identification of ADK Genes in the Pepper Genome and Subcellular Localization Analysis

The genome of ‘Zunla-1’, sequenced and assembled in China, served as the primary reference genome for this study (accession no. PRJcA025503). Genome sequences of *Oryza sativa* (rice), *Solanum lycopersicum* (tomato), and *Solanum tuberosum* (potato) were retrieved from the NCBI database (https://www.ncbi.nlm.nih.gov/, accessed on 2 February 2025) for comparative analysis. To annotate *CaADK* genes in the pepper genome, the full protein sequences of *ADKs* in *Arabidopsis* were obtained from the TAIR database (https://www.arabidopsis.org/, accessed on 2 February 2025). Sequences of *Arabidopsis* served as queries for a BLAST searches (E-value < 1 × 10^−10^) against the pepper genome. Pfam domain databases (https://www.ebi.ac.uk/interpro/, accessed on 13 February 2025) were employed to construct a genome-wide protein domain model for pepper [40,41].

The physicochemical properties of pepper *ADK* gene family members were predicted using the ExPASy technologies server (http://web.expasy.org/, accessed on 23 February 2025), and the subcellular distribution was analyzed using WoLF PSORT (http://wolfpsort.org/, accessed on 23 February 2025) [40,42].

### 4.2. Chromosomal Localization, Synteny, and Phylogenetic Analysis

The chromosomal locations of *CaADK* family genes were mapped using TBtools, and intra-genomic synteny analysis was conducted with MCScanX [43,44]. Amino acid (aa) sequences of *CaADKs* and *ADK* genes from other species were aligned via the MUSCLE algorithm. Phylogenetic inference was performed in MEGA X, with all major clades having bootstrap support > 95% (1000 replicates) [45]. Phylogenetic trees were visualized and annotated using iTOL (https://itol.embl.de/, accessed on 24 February 2025) [46].

### 4.3. Motif, Gene Structure, and Conserved Domain Analysis

Coding sequence (CDS) and gff3 format files of *Capsicum annuum* were downloaded from the pepper genome database (http://www.bioinformaticslab.cn/PepperBase/, accessed on 24 February 2025) [47]. The intron-exon architectures were visualized using TBtools software. Conserved protein motifs were identified using the online software MEME suite version 5.5.8 [48]. In MEME (Multiple Expectation Maximization for Motif Elicitation) analysis, the maximum number of motifs was set to 10, and the occurrences of a single motif were set to zero or one per sequence. These were visualized using “Visualize Motif Pattern” (from the MEME suite) in TBtools.

### 4.4. Protein Secondary and Tertiary Structure Analysis

PRABI was used to analyze the secondary structure of CaADK proteins (https://prabi.ibcp.fr/htm/site/web/app.php/home, accessed on 24 February 2025), and SWISS-MODEL (https://swissmodel.expasy.org/, accessed on 25 February 2025) was employed for the protein tertiary structure analysis [49].

### 4.5. Cis-Acting Element Analysis and Gene Ontology Analysis

The 2-kb sequences upstream of the *CaADK* family genes were selected for *cis*-acting element analysis using the online tool PlantCARE with default parameters [50]. A gene ontology (GO) analysis of the *CaADK* genes was conducted with default parameters at the DAVID website (http://david.ncifcrf.gov, accessed on 26 February 2025) [51].

### 4.6. Tissue-Specific Expression

To profile tissue-specific expression patterns of *CaADK* genes, the transcriptome annotation files of roots, stems, leaves, buds, flowers, and fruit development stages of pepper ‘Zunla-1’ were obtained through the GEO database (https://www.ncbi.nlm.nih.gov/geo/query/acc.cgi?acc%20=%20GSE45037, accessed on 24 February 2025). Tissue-specific expression data were analyzed using TBtools (v1.120).

### 4.7. Transcriptome Data Analysis of ADK Gene Family in Pepper

Publicly available transcriptome data for ‘Zunla-1’ was downloaded from the NCBI GEO database (accession numbers GSE132824 and GSE149037). Gene expression data for *CaADK* genes was collected in leaves from 0 to 24 h (sampled at 1, 3, 6, 12, and 24 h) after different abiotic stresses, such as cold stress (10 °C), heat stress (40 °C), salt stress (400 mM NaCl), and osmotic stress (400 mM mannitol), as well as different phytohormone treatments, including SA (5 mM sodium salicylate) and ET (5 mM ethephone). The gene expression level of *CaADKs* was calculated by the log_2_ FPKM algorithm, and expression data were analyzed using TBtools.

### 4.8. RNA Extraction, cDNA Synthesis, and RT-qPCR

The test materials (‘Hangjiao-1’ pepper) were exposed to cold stress (4 °C for 0, 4, 8, and 24 h) and salt stress (150 mM NaCl for 0, 4, 8, and 24 h) at the College of Life Sciences, South China Agricultural University (Guangzhou, Guangdong, China). Control (CK) seedlings were maintained under normal conditions. Following stress treatment, the materials were rapidly frozen in liquid nitrogen for further experiments. RNA integrity was evaluated by 1% agarose gel electrophoresis, and the RNA concentration and purity were measured using a spectrophotometer Thermo NanoDrop One (Thermo Fisher Scientific, Waltham, MA, USA).

cDNA was synthesized by reverse transcription using HiScript^®^ II Q RT SuperMix with a qPCR sample kit (Vazyme Biotech Co., Ltd., Nanjing, China), then diluted ten-fold and stored at −20 °C. RT-qPCR was conducted in 20 µL volume containing 2 × SYBR qPCR Master Mix (10.0 μL), upstream and downstream primers (10 μmol∙L^−1^, 0.4 μL each), cDNA template (1.0 μL), and ddH2O (8.2 μL). Thermal cycling conditions included initial denaturation at 95 °C for 30 s; 40 cycles of 95 °C for 10 s (denaturation) and 60 °C for 22 s (annealing/extension); followed by melt curve analysis at 95 °C for 25 s, 60 °C for 60 s, and 95 °C for 7 s. Actin was used as the internal control. The 2^−∆∆CT^ method was used to analyze relative expression levels [52].

### 4.9. PPI Network Prediction Analysis of ADK Genes

The STRING online website (https://cn.string-db.org/, accessed on 27 February 2025) was used to predict the PPI relationships with default parameters [53], and Cytoscape v3.9.1 was used to construct the interaction network [54].

## 5. Conclusions

**Figure 11:**
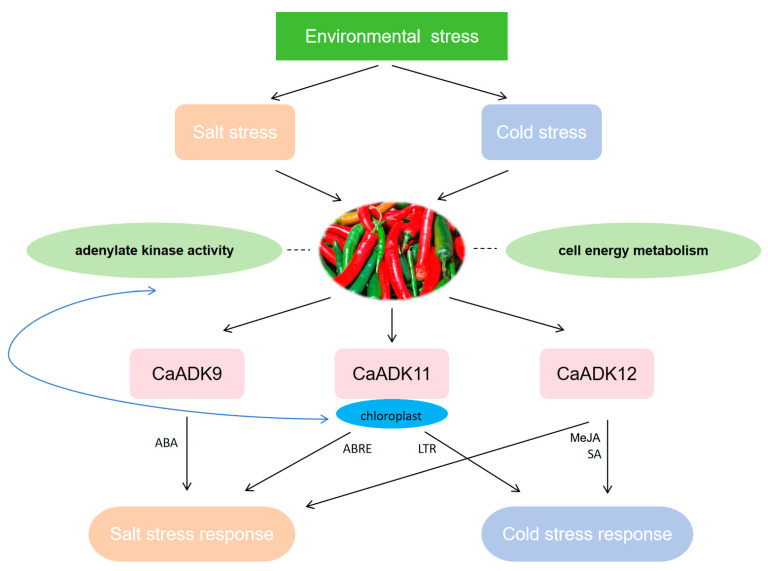
The diagram of *ADK* genes regulating stress responses in pepper.

In this study, we systematically identified 15 adenylate kinase genes (*CaADKs*) in the pepper (*Capsicum annuum* L.) genome, representing the first comprehensive characterization of the *ADK* family in this crop. Our analysis enriches the current understanding of *ADK* gene family evolution and function in Solanaceous plants, complementing existing studies in tomato and potato. Phylogenetic and collinearity analyses revealed that pepper ADKs share a significantly higher level of homology with other Solanaceous crops, including tomato and potato, providing evidence supporting monocot-dicot divergence in the *ADK* gene family. Through integrated approaches, including subcellular localization prediction, *cis* element profiling, stress-responsive expression dynamics, and RT-qPCR validation, we identified distinct roles of specific members: *CaADK9*, characterized by upregulation under salt stress and presence of abscisic acid-responsive promoter elements, mediates salt stress adaptation; *CaADK11* and *CaADK12*, which exhibit significant downregulation under both low-temperature and salt stress, act as critical regulators of combined stress tolerance (schematically summarized in Figure 11).

## Figures and Tables

**Table 1:** Information of *ADK* family members in pepper.

Gene ID	Name	Chr	Start	End	Subcellular	AA	II	GRAVY	MW (Da)	PI
*Capana01g000752*	*CaADK1*	1	15523773	15526802	Nuclear	226	36.23	−0.464	25,452.25	7.59
*Capana01g001211*	*CaADK2*	1	36924721	36925447	Cytoplasmic	125	61.98	−0.431	14,101.31	6.09
*Capana01g003380*	*CaADK3*	1	225341827	225345336	Cytoplasmic	209	35.56	−0.291	22,892.08	5.3
*Capana02g003629*	*CaADK4*	2	163094406	163095880	Chloroplast	264	38.06	−0.215	29,387.73	6.96
*Capana03g001368*	*CaADK5*	3	24852841	24856942	Cytoskeletal	233	41.79	−0.29	25,466.41	6.91
*Capana03g002276*	*CaADK6*	3	57187892	57188440	Nuclear	119	40.43	−0.355	13,409.26	4.97
*Capana03g002509*	*CaADK7*	3	78776948	78779833	Mitochondrial	248	46.15	−0.466	28,238.46	7.63
*Capana04g001180*	*CaADK8*	4	36386958	36387557	Cytoplasmic	152	43.75	0.025	17,260.02	5.91
*Capana04g002894*	*CaADK9*	4	215636406	215639140	Nuclear	251	52.09	−0.33	28,302.5	6.26
*Capana05g001537*	*CaADK10*	5	147669252	147669866	Cytoplasmic	127	31.04	−0.012	14,598.09	5.92
*Capana06g001357*	*CaADK11*	6	29729067	29732546	Chloroplast	237	54.74	−0.278	25,989.66	6.84
*Capana09g000061*	*CaADK12*	9	1398616	1404683	Cytoplasmic	536	36.79	−0.318	59,842.7	5.79
*Capana09g001871*	*CaADK13*	9	213514351	213519615	Chloroplast	333	40.22	−0.152	36,395.87	8.13
*Capana11g001398*	*CaADK14*	11	166349615	166351052	Cytoplasmic	112	30.56	0.302	12,137.1	4.67
*Capana12g001687*	*CaADK15*	12	145918582	145930123	Mitochondrial	280	45.31	−0.272	31,145.88	6.54
Average	\	\	\	\	\	245.23	43.11	−0.30	27,324.78	6.53

**Table 2:** Information regarding ADK motifs in pepper.

Motif	Motif Consensus
Motif 1	GGPGSGKGTQCERJAKLFGLPHISTGDLLRQEJKSGSELGKKIAEIMNZG
Motif 2	GEKGFJLDGFPRSKIQAEILE
Motif 3	GVDPDLVLNLKCPEEILVKRVLGRRLYP
Motif 4	DDNEDTVRERLKVYMESSLPVEEYYRKKGKLLEFDAAGGIPEVWZKLLAA
Motif 5	KLVPEEVIFGLLSKRLEEGYC
Motif 6	SFADDGKRVKVCVQGSLGEGALAGMPLQLAESRKILEFMDWGDYGALGTF
Motif 7	KGRGVQWVIM
Motif 8	EFLCRGTYEINTSRQAEGDHFRPSSIMDDVSRKNLQVH
Motif 9	QDDLFILVAFQNAVENCIIDVIHRE
Motif 10	TGKIYHLKYSPPETEEI

## Data Availability

The original contributions presented in the study are included in the article and Supplementary Materials, further inquiries can be directed to the corresponding authors.

## Supplementary Materials

The following supporting information can be downloaded at: https://www.mdpi.com/article/10.3390/ijms262010213/s1.

## References

[1] Fujisawa K. (2023). Regulation of Adenine Nucleotide Metabolism by Adenylate Kinase Isozymes: Physiological Roles and Diseases. Int. J. Mol. Sci..

[2] Pradet A., Raymond P. (1983). Adenine nucleotide Ratios and Adenylate Energy Charge in Energy Metabolism. Annu. Rev. Plant Physiol..

[3] Park J., Gupta R.S. (2012). Adenosine Meta Bolism, Adenosine Kinase, and Evolution.

[4] Dzheia P., Kal’venas A., Toleĭkis A., Prashkiavichius A. (1986). The role of adenylate kinase in the regulation of the rate and effectiveness of energy transfer from mitochondria to hexokinase in vitro. Biokhimiia.

[5] Lange P.R., Geserick C., Tischendorf G., Zrenner R. (2008). Functions of chloroplastic adenylate kinases in Arabidopsis. Plant Physiol..

[6] Regierer B., Fernie A.R., Springer F., Perez-Melis A., Leisse A., Koehl K., Willmitzer L., Geigenberger P., Kossmann J. (2002). Starch content and yield increase as a result of altering adenylate pools in transgenic plants. Nat. Biotechnol..

[7] Arora K., Brooks C.L. (2007). Large-scale allosteric conformational transitions of adenylate kinase appear to involve a population-shift mechanism. Proc. Natl. Acad. Sci. USA.

[8] Maragakis P., Karplus M. (2005). Large amplitude conformational change in proteins explored with a plastic network model: Adenylate kinase. J. Mol. Biol..

[9] Birkenhead K., Walker D., Foyer C. (1982). The intracellular distribution of adenylate kinase in the leaves of spinach, wheat and barley. Planta.

[10] Boonrueng C., Tangpranomkorn S., Yazhisai U., Sirikantaramas S. (2016). Molecular cloning, subcellular localization and characterization of two adenylate kinases from cassava, *Manihot esculenta* Crantz cv. KU50. J. Plant Physiol..

[11] Kawai M., Kidou S.I., Kato A., Uchimiya H. (1992). Molecular characterization of cDNA encoding for adenylate kinase of rice (*Oryza sativa* L.). Plant J..

[12] Carrari F., Coll-Garcia D., Schauer N., Lytovchenko A., Palacios-Rojas N., Balbo I., Rosso M., Fernie A.R. (2005). Deficiency of a Plastidial Adenylate Kinase in *Arabidopsis* Results in Elevated Photosynthetic Amino Acid Biosynthesis and Enhanced Growth. Plant Physiol..

[13] Weretigk E.A., Alexander K.J., Drebensteat M., Ssnider J.D., Summers P.S., Mofratt B.A. (2001). Maitaning Mtlyation Actvties Durng Salt Stess, the Ivovement of Adenosine Kinase 1. Plant Physiol..

[14] Gai J.T., Zhao T.J., Li Y., Gai J.Y. (2013). Cloning and Expression Analysis of an Adenylate Kinase Gene *GmADK* in Soybean. Acta Agron. Sin..

[15] Gong P., Zhang J., Li H., Yang C., Zhang C., Zhang X., Khurram Z., Zhang Y., Wang T., Fei Z. (2010). Transcriptional profiles of drought-responsive genes in modulating transcription signal transduction, and biochemical pathways in tomato. J. Exp. Bot..

[16] Zhou S., Wei S., Boone B., Levy S. (2007). Microarray analysis of genes affected by salt stress in tomato. Afr. J. Environ. Sci. Technol..

[17] Baenas N., Belović M., Ilic N., Moreno D.A., García-Viguera C. (2019). Industrial use of pepper (*Capsicum annum* L.) derived products: Technological benefits and biological advantages. Food Chem..

[18] Jarret R.L., Barboza G.E., Costa Batista F.R., Berke T., Chou Y., Hulse-Kemp A., Ochoa-Alejo N., Pasquale T., Veres A., Carrizo Garcia C. (2019). Capsicum—An Abbreviated Compendium. J. Amer. Soc. Hort. Sci..

[19] Bae Y., Lim C.W., Lee S.C. (2021). Differential Functions of Pepper Stress-Associated Proteins in Response to Abiotic Stresses. Front. Plant Sci..

[20] Särkinen T., Huang S., Li X., Wang X., Soltis D.E., Soltis P.S., Zhang C. (2015). The evolutionary history of Solanaceae inferred from nuclear transcriptomes and genomes. Nature.

[21] Chen H., Jiang L., Chen S., Zhang X., Zhu N., Wei P., Zhou C. (2020). Adk1 Overexpression and Sodium Citrate Feeding Enhanced S-adenosylmethionine Synthesis in Yeast. J. Agri. Sci. Technol..

[22] Igamberdiev A.U., Kleczkowski L.A. (2006). Equilibration of adenylates in the mitochondrial intermembrane space maintains respiration and regulates cytosolic metabolism. J. Exp. Bot..

[23] Luo Z., Liu Z., Liu C., Li L., Xu D. (2023). Bioinformatics analysis of rice ADK gene family. Mol. Plant Breed..

[24] Yang L., Cao H., Zhang X., Gui L., Chen Q., Qian G., Xiao J., Li Z. (2021). Genome-Wide Identification and Expression Analysis of Tomato ADK Gene Family during Development and Stress. Int. J. Mol. Sci..

[25] Wang Y., Lei Y., Xu J., Wei Z., Wei J., Min X. (2022). Identification and analysis of ADK gene family members in alfalfa (*Medicago sativa*). Caoye Kexue.

[26] Xu G., Guo C., Shan H., Kong H. (2012). Divergence of duplicate genes in exon–intron structure. Proc. Natl. Acad. Sci. USA.

[27] Peters S.A., Bargsten J.W., Szinay D., van de Belt J., Visser R.G., Bai Y., de Jong H. (2012). Structural homology in the Solanaceae: Analysis of genomic regions in support of synteny studies in tomato, potato and pepper. Plant J..

[28] Li X., Lyu C., Song J., Lu Y., Zeng F., Lu L., Li L. (2023). Identification and Expression Analysis of Adenylate Kinase Gene Family in Potato. Horticulturae.

[29] Lee S.C., Luan S. (2012). ABA signal transduction at the crossroad of biotic and abiotic stress responses. Plant Cell Environ..

[30] Hong J.H., Seah S.W., Xu J. (2013). The root of ABA action in environmental stress response. Plant Cell Rep..

[31] Zhang J., Jia W., Yang J., Ismail M. (2006). Role of ABA in integrating plant responses to drought and salt stresses. Field Crops Res..

[32] Peterson T.A., Nieman R.H., Clark R.A. (1987). Nucleotide Metabolism in Salt-Stressed *Zea mays* L. Root Tips: I. Adenine and Uridine Nucleotides. Plant Physiol..

[33] Lantzouni O., Alkofer A., Falter-Braun P., Schwechheimer C. (2020). GROWTH-REGULATING FACTORS Interact with DELLAs and Regulate Growth in Cold Stress. Plant Cell.

[34] Saleem M., Fariduddin Q., Janda T. (2021). Multifaceted Role of Salicylic Acid in Combating Cold Stress in Plants: A Review. J. Plant Growth Regul..

[35] Wan S.-B., Tian L., Tian R.-R., Pan Q.-H., Zhan J.-C., Wen P.-F., Chen J.-Y., Zhang P., Wang W., Huang W.-D. (2009). Involvement of phospholipase D in the low temperature acclimation-induced thermotolerance in grape berry. Plant Physio. Biochem..

[36] Ding F., Wang X., Li Z., Wang M. (2022). Jasmonate Positively Regulates Cold Tolerance by Promoting ABA Biosynthesis in Tomato. Plants.

[37] Nie G., Zhou J., Jiang Y., He J., Wang Y., Liao Z., Appiah C., Li D., Feng G., Huang L. (2022). Transcriptome characterization of candidate genes for heat tolerance in perennial ryegrass after exogenous methyl Jasmonate application. BMC Plant Biol..

[38] Hampp R., Goller M., Ziegler H. (1982). Adenylate levels, energy charge, and phosphorylation potential during dark-light and light-dark transition in chloroplasts, mitochondria, and cytosol of mesophyll protoplasts from *Avena sativa* L.. Plant Physiol..

[39] Stitt M., Lilley R.M., Heldt H.W. (1982). Adenine Nucleotide Levels in the Cytosol, Chloroplasts, and Mitochondria of Wheat Leaf Protoplasts 1. Plant Physiol..

[40] Artimo P., Jonnalagedda M., Arnold K., Baratin D., Csardi G., de Castro E., Duvaud S., Flegel V., Fortier A., Gasteiger E. (2012). ExPASy: SIB bioinformatics resource portal. Nucleic Acids Res..

[41] Xu Q., Dunbrack R.L. (2012). Assignment of protein sequences to existing domain and family classification systems: Pfam and the PDB. Bioinformatics..

[42] Horton P., Park K.J., Obayashi T., Fujita N., Harada H., Adams-Collier C.J., Nakai K. (2007). WoLF PSORT: Protein localization predictor. Nucleic Acids Res..

[43] Chen C., Chen H., Zhang Y., Thomas H.R., Frank M.H., He Y., Xia R. (2020). TBtools: An Integrative Toolkit Developed for Interactive Analyses of Big Biological Data. Mol. Plant.

[44] Wang Y., Tang H., Debarry J.D., Tan X., Li J., Wang X., Lee T.H., Jin H., Marler B., Guo H. (2012). MCScanX: A toolkit for detection and evolutionary analysis of gene synteny and collinearity. Nucleic Acids Res..

[45] Kumar S., Stecher G., Li M., Knyaz C., Tamura K. (2018). MEGA X: Molecular Evolutionary Genetics Analysis across Computing Platforms. Mol. Biol. Evol..

[46] Letunic I., Bork P. (2021). Interactive Tree Of Life (iTOL) v5: An online tool for phylogenetic tree display and annotation. Nucleic Acids Res..

[47] Qin C., Yu C., Shen Y., Fang X., Chen L., Min J., Cheng J., Zhao S., Xu M., Luo Y. (2014). Whole-genome sequencing of cultivated and wild peppers provides insights into *Capsicum* domestication and specialization. Proc. Natl. Acad. Sci. USA.

[48] Bailey T.L., Williams N., Misleh C., Li W.W. (2006). MEME: Discovering and analyzing DNA and protein sequence motifs. Nucleic Acids Res..

[49] Waterhouse A., Bertoni M., Bienert S., Studer G., Tauriello G., Gumienny R., Heer F.T., de Beer T.A.P., Rempfer C., Bordoli L. (2018). SWISS-MODEL: Homology modelling of protein structures and complexes. Nucleic Acids Res..

[50] Lescot M., Déhais P., Thijs G., Marchal K., Moreau Y., Van de Peer Y., Rouzé P., Rombauts S. (2002). PlantCARE, a database of plant *cis*-acting regulatory elements and a portal to tools for in *silico* analysis of promoter sequences. Nucleic Acids Res..

[51] Dennis G., Sherman B.T., Hosack D.A., Yang J., Gao W., Lane H.C., Lempicki R.A. (2003). DAVID: Database for Annotation, Visualization, and Integrated Discovery. Genome Biol..

[52] Livak K.J., Schmittgen T.D. (2001). Analysis of relative gene expression data using real-time quantitative PCR and the 2^−∆∆CT^ Method. Methods.

[53] Szklarczyk D., Gable A.L., Nastou K.C., Lyon D., Kirsch R., Pyysalo S., Doncheva N.T., Legeay M., Fang T., Bork P. (2021). The STRING database in 2021: Customizable protein-protein networks, and functional characterization of user-uploaded gene/measurement sets. Nucleic Acids Res..

[54] Shannon P., Markiel A., Ozier O., Baliga N.S., Wang J.T., Ramage D., Amin N., Schwikowski B., Ideker T. (2003). Cytoscape: A software environment for integrated models of biomolecular interaction networks. Genome Res..

