# Supplementary material for: Genome-Wide Identification and Expression Analysis of Adenylate Kinase Family Members in Pepper Under Abiotic Stress
Source: Int J Mol Sci. 2025 Oct 21;26(20):10213. doi: 10.3390/ijms262010213 (PMC12564395; doi:10.3390/ijms262010213)
Supplement: Supplementary file 1 [file ijms-26-10213-s001.zip › Table S1.pdf]

Table S1 The protein sequences of the ADK proteins from pepper species and *Arabidopsis*, rice, potato and tomato

Pepper (*Capsicum annuum*)

>CaADK1

MLCGALLSAHELFVESDRGLAKQKKVKVVFVIGGPGSGKGTQCQRIAQQFGYTHLSVGEL
LRQEINSGSETGSMIHSIMKEGKLVPSDVTVRLLQQAMQGIDNDKFLIDGFPRNEENVKT
FENLTKMEPEFVLYLDCPQDEMERRLLSRNEGREDDNIDTIRKRFKVFMESTLPAVEYYE
SKGKIRKVDAGKSIDDVFESIKAIFSQGKDNNKVPPSKNKCKCLIL

>CaADK2

MHIAAEDLPRAEIAAGSKNRMQAKKSMDKGKLAPDEIVVTMVKEQLNGPDSQEKGWLLDG
YPRSSSQAITLKEFGLQPDLHIHLEVPKEILVERVVGRRLDLITVKIYHLKYSPLETEEI
ESKLT

>CaADK3

MGTVVDSANQGAGSLTINKKVTVVFVLGGPGSGKGTQCANIVENFGYTHLSAGDLLRAEI
KSGSENGTMISNMIKEGKIVPSEVTIKLLQRAIQENGNDKFLIDGFPRNEENRAAFELVT
GIEPEFVLFFDCPEEEMEKRLLGRNQGREDDNIETIRKRFKVYMESSLPVIEYYNSKGKV
RKIDAAKPVGEVFEAVKAVFAPADEKVAA

>CaADK4

MAVISFLGVSARQLTIRASRKCLRAYGSAAAVQLDYEYEEEMEESAVSAKGRGVQWLIMG
HPMTQRHVYARWLSKLLDAPHISMGSLVRQELHPRSTLYNKIASAVNEGKLVPEDVVFGL
LSKRLEEGYCRGESGFILDGIPRTIFQAEILDKVVDIDLVLNLKCSDCYVPKNDTSNGIY
AAEDQLLTRGNLMSSRVMDGAYREKQNALAEQIKPLEEYYRKQKKLLDFQVAGGPAETWQ
GLLAALQLQHMRSSVGSTQLSAGC

>CaADK5

MSTSVNLEDVPSESIMSELLRRMKCSSKPDKRLILIGPPGSGKGTQSPIIKDEYCLCHLA
TGDMLRAAVAAKTPLGIKAKEAMDKGELVSDDLVVGIIDEALKKPSCQKGFILDGFPRTV
VQAEKLDVMLQNRGTKVDKVLNFAIDDAILEERITGRWIHPASGRSYHTKFAPPKVPGTD
DVTGEPLIQRKDDTAAVLKSRLEAFHRQTEPVIDYYAEKPPQAVTAEVKTALS

>CaADK6

MSCSEGLVPGRGVQWVIMGDPIAQRHVGKSGFVLDGIPRSKIQDEILDKTVDIDLVLNLK
CAEDLVSKKEKNPRLYPSLEFLCTGTYEINTSFYSEGDHFRPSSIMDDVSRKNLQVYEE

>CaADK7

MWRRLTSLSHLRQIRRADELKICQAFCTDITKPPVEAESHYRRNSPFVAFVLGGPGSGKG
TQCLKIAETFGFEHIGAGDLLRKEMYSGSENGDMIQKLMKEGSIAPSEVTVKLIKKAIES
AENRKFLIDGFPRSEENRMAYERIVGAEPNFVLFFDCPEEVMVKRVLNRNEGRVDDNEHT
VKERLKVYKGTTLPVVNHYAKKGKLYKVDGTGTQEEIFERVVDITDTLQKQVIGIEDVEC
AVEKYMRM

>CaADK8

MPLCFQPSVDIKMVLSHMMNRFSNYTALSAEVSAKNQTSLQEDNTFWFLNNFFLQVLPEF
FRVEVFAKLNSAIGKLEGGHFKPLSIMDDVSRKNLQMHTEQVKPLEEYYMKQIKFLDFQV
AGGPVEILQGLLVALHLQHMDAVSSAQLTAGC

>CaADK9

MALLSRMRAAAKPLMRRRYGSAAAAQLADYDDYDEYDEECCEKNRRCLMEESEGSVPTRG

VQWVIMGDPMAKRHVYAQWLAKLLDVPHISMGSLVRQELHPRSTLYKQIADAVNQGKLVP
EEVIFGLLSKRLEEGYCRGESGFILDGIPRSKIQAEILDKTVDIDLVLNLKCAEDLMSKK
EKSPRLYPSLEFLCRGTSGINTSRQAEGSHFRPSSIMDDVSRKNLHVHAEQASFDSHPDL
HISLLLLVLVS
>CaADK10
MALLSRLRAAEYPLIQIIHHELHPFSTLYKQIADVVSQGKLFLEEVIFGLLSKRLEEGYC
RGEIGFILGGIPRSKIKDEIPDKTVDTDLVLNLKCAEDLVSKKDKSTRLYPLLEFLFMEI
YEINTSR
>CaADK11
MALCCSLSFPTVSTKPNKSSSSPICSSLQLTFTAHLPFSKSSSFYPNNTLLQTQCRGNPS
TNGPSFLVVGSAKKQEPLRVMISGAPASGKGTQCELITKKYDLVHVAAGDLLRAEIAAGT
ENGRRAKEYMDKGQLVPNEIVVTVELLVRPDSQEKGWLLDGYPRSSSQALALKEFGFQPD
LFILLEVPEEILVERVVGRRLDPVTGRIYHLKYSPPETDEIAARLTQRFDDTEEKAS
>CaADK12
MKVICGRSEPLKVMISGAPASGKGTQCELIVQKFGLVHISTGDLLRAELSAGTDIGNKAK
EYMNSGRLVPDEIVTAMVTARLSREDAKEKGWLLDGYPRKLAQAESLERLNIRPDIYIVL
DVPDEILIDRCVGRKLDPLTGKIYHVTHFPPETEDIKARLITRPDDTEEKVKSRLQIYKQ
NAEAILPVYSNIMKKVDGNSDKDAVFEEIDSLLSRVQKEEQDARKSEAAVISSTRADITS
LNKDWRGIPTRLNNIPHSREIREYFYTDVLQATQRAVNDGKTRLKIEISIPELNPSMDVY
RIGTLMELIRVLALSFADDGKRVKVCVQGSMGEGALAGMPLQLAGSRKILEFMDWGDYGA
LGTFVNIGSIGGKEVGEQDDLFILVAPQNAVGNCIIDVIHREPVADMKAMTDAAGNRPVI
LVNPKLKDLPGSSGIMQTMGRDKRLEYAASFEICYQFRLLYYAGTQYPIMGALRMSYPYP
YELYKRVDESPGKEKYISLATFAKRPSVDEMNDAFEGKPRYATDHEPELVINSYTC
>CaADK13
MAAMIRLFRSSSSSSYSRYFSTAAVSETVKSQSYPRNPVTTNVEPKGKTVQWVFLGCPGV
GKGTYASRLSTLLGVPHIATGDLVRDELKSSGSLSKQGKTVQWVFLGCPGVGKGTYARRL
STLLGVPHIATGDLVRDELKILGPLLKQLAEIVNQGKLVSDEIILNLLSKRLENGEAKGE
AGFILDGFPRTVRQAEILTEVTDIDLVVNLKLPESVLVEKCLGRRICSECGKNFNVASID
VAGENGAPRITMAPLNPPTQCISKLITRADDTEDIVKERLRIYWDKSQPVEDFYRSQGKL
LEFDLPGGIPESWPKLLEVLNLDEQEHKLSAAA
>CaADK14
MKLIRVLALSFADDGKRVKVCVQGSLGKGALAGMPLQLAESQKILEFMDWGDYDTLGTFV
NSGSIGMTEIKIACGKEVAGQDDLFILVAFQNAVENCIIDVIHRELFSDLPS
>CaADK15
MAVWSRAVVRWRCRTTNLTRGFCYKPSEDNVKVVVSPTNCLETKGRNVQWVFLGCPGVGK
GTYAARLSKLLGVPHIATGDLVRQQLSSHGPLASQLADIVNQGQLISDEIVIDLLSKSLE
AGEAKGETGFILDGFPRTMRQAEILEGVTDIDLVINLKLREDALIAKCLGRRTCSECGGN
YNVACIDMKGEDGKTRMYMPPLLPPPHCETKLITRSDDTENVVKERLRIYHEMSQPVEDF
YRMRGKLLEFDLPGGIPESWPKLLHALNIYDDEDKKSAAA

***Arabidopsis* (*Arabidopsis thaliana*)**
>At5g50370
MATSSAASVDMEDIQTVDLMSELLRRMKCASKPDKRLVFIGPPGSGKGTQSPVIKDEFCL
CHLSTGDMLRAAVAAKTPLGVKAKEAMDKGELVSDDLVVGIMDEAMNRPKCQKGFILD

GFPRTVTQAEKLDEMLNRRGAQIDKVLNFAIDDSVLEERITGRWIHPSSGRSYHTKFAPPK
VPGVDDLTGEPLIQRKDDNADVLRSRLDAFHKQTQPVIDYYAKKENLVNIPAEKAPEEVT
KVVKKVVST
>AT5g63400
MATGGAAADLEDVQTVDLMSELLRRLKCSQKPDKRLIFIGPPGSGKGTQSPVVKDEYCLC
HLSTGDMLRAAVASKTPLGVKAKEAMEKGELVSDDLVVGIIDEAMNKPKCQKGFILDGFP
RTVTQAEKLDEMLKRRGTEIDKVLNFAIDDAILEERITGRWIHPSSGRSYHTKFAPPKTPGV
DDITGEPLIQRKDDNADVLKSRLAAFHSQTQPVIDYYAKKAVLTNIQAEKAPQEVTSEVK
KALS
>AT5g47840
MTGCVNSISPPPVTLYRHRASPSRSSFSLSGDALHSLYRHRRVSRSPSIIAPKFQIVAAEKSEP
LKIMISGAPASGKGTQCELITHKYGLVHISAGDLLRAEIASGSENGRRAKEHMEKGQLVPD
EIVVMMVKDRLSQTDSEQKGWLLDGYPRSASQATALKGFGFQPDLFIVLEVPEEILIERVV
GRRLDPVTGKIYHLKYSPPETEEIAVRLTQRFDDTEEKAKLRLKTHNQNVSDVLSMYDDIT
IKIEGNRSKEEVFAQIDSSLSELLQERNTAPSSLLS
>AT5g35170
MASLSLSSAHFSSTSSSSRSSISTSSLSPSSTSLPLLQSPIRRRYRSLRRRLSFSVIPRRTSRSFS
TSNSQIRCSINEPLKVMISGAPASGKGTQCELIVHKFGLVHISTGDLLRAEVSSGTDIGKRA
KEFMNSGSLVPDEIVIAMVAGRLSREDAKEHGWLLDGFPRSFAQAQSLDKLNVKPDIFILL
DVPDEILIDRCVGRRLDPVTGKIYHIKNYPPESDEIKARLVTRPDDTEEKVKARLQIYKQNS
EAIISAYSDVMVKIDANRPKEVVFEETQTLLSQIQLKRMIKTDKASPVQDKWRGIPTRLNN
IPHSRDIRAYFYEDVLQATIRSIKDGNTRLRVDINIPELNPEMDVYRIGTLMELVQALALSFA
DDGKRVKVCVQGSMGEGALAGMPLQLAGTRKILEYMDWGDDETLGTFVKLGAIGGKE
VDEEDDMFILVAPQNAVGNCIIDDLQAMTTAAGKRPVVLINPRLKDLPASSGIMQTMGRE
QRLEYALTFDNCYVFRLLYYLGTQYPIMGALRMSYPYRYELYKRVNEENGKEKYVLLATY
AERPTPEQIDDAFSGKSRDQSKKASGIWGFLSSVFS
>AT3g01820
MAWLSRVRGVSPVTRLAAIRRSFGSAAALEFDYDSDDEYLYGDDRRLAEPRLGLDGSGP
DRGVQWVLMGAPGAWRHVFAERLSKLLEVPHISMGSLVRQELNPRSSLYKEIASAVNERK
LVPKSVVFALLSKRLEEGYARGETGFILHGIPRTRFQAETLDQIAQIDLVVNLKCSEDHLVN
RNETALPQQEFLGSMLHSPVAINARRESVGVYAQEVEEYYRKQRKLLDFHVGGATSADT
WQGLLAALHLKQVNLTTSQKLTL
>AT2g39270
MAVSHRLLRPATTTIKNTFSSLFIRSLSSSSSGSSLDPKIDLEEAAAQLGKSSSTSTSPYKGRN
FHWVFLGCPGVGKGTYASRLSSLLGVPHIATGDLVREELSSSGLLSSQLKELVNHGKLVPD
EFIISLLSKRLQAGKDKGESGYILDGFPRTVTQAEILEGVTNIDLVINLKLREEALLAKCLGR
RICSECGGNYNVACIDIKGDDDTPRMYMPPLLPPPNCESKLISRADDTEEVVKERLRIYNK
MTQPVEEFYKKRGKLLEFELPGGIPESWARLLRALHLEDDKQSAIA
>AT2g37250
MARLVRVARSSSLFGFGNRFYSTSAEASHASSPSPFLHGGGASRVAPKDRNVQWVFLGCP
GVGKGTYASRLSTLLGVPHIATGDLVREELASSGPLSQKLSEIVNQGKLVSDEIIVDLLSKR
LEAGEARGESGFILDGFPRTMRQAEILGDVTDIDLVVNLKLPEEVLVDKCLGRRTCSQCGK
GFNVAHINLKGENGRPGISMDPLLPPHQCMSKLVTRADDTEEVVKARLRIYNETSQPLEE
YYRTKGKLMEFDLPGGIPESWPRLLEALRLDDYEEKQSVAA

Rice (*Oryza sativa*)

>Os12t0236400

MAANLEDVPSMELMTELLRRMKCSSKPDKRVILVGPPGCGKGTQSPLIKDEFCLCHLATG
DMLRAAVAAKTPLGIKAKEAMDKGELVSDDLVVGIIDEAMKKTSCQKGFILDGFPRTVVQ
AQKLDEMLAKQGTKIDKVLNFAIDDAILEERITGRWIHPSSGRSYHTKFAPPKTPGLDDVT
GEPLIQRKDDTAAVLKSRLEAFHVQTKPVIDYYTKKGIVANLHAEKPPKEVTVEVQKALS

>Os11t0312220

MAAAANLEDVPSMDLMNELLRRMKCSSKPDKRLILVGPPGSGKGTQSPIIKDEYCLCHLA
TGDMLRAAVAAKTPLGVKAKEAMDKGELVSDDLVVGIIDEAMKKPSCQKGFILDGFPRT
VVQAQKLDEMLEKKGTKVDKVLNFAIDDSILEERITGRWIHPSSGRSYHTKFAPPKVPGV
DDVTGEPLIQRKDDTAEVLKSRLEAFHKQTEPVIDYYSKKALVANLHAEKPPKEVTAEVQ
KVLS

>Os08t0288200

VHISTGDLLRAEVSSGTEIGKKAKEYMDNGMLVPDQVVTDMVVSRLSQPDVRERGWLL
DGYPRSYAQAQSLESMKIRPDIFIVLEVPDDILIDRCVGRRLDPETGKIYHIKNFPPENDEVS
ARLVTRSDDTFEKVKSRLDTYKQNSEAVIPTYSDLLNQIDGNRQVEVVFNEIDSLLQKICE
NASFNMLAKTNGKPQDSKDTTASKNEFRGIPTRLNNIPHSREIRKYFYNDVLVATRHAVED
KKTRLQIDINIPELNPEMDVYRIGTLMELVRELSLSFADDGKRVKVCVQGSMGQGAFAGIP
LQLAGTRKILEIMDWGEYGAKGTFINFGAVGASEVDKEDDMFILIAPQNAVGNCIIDDMK
AMTDAAGDRPVILVNPRLKDMPGSSGVMQTMGRDMRLKYAASFETCYSFRLLFYAGSFY
PIMGALRMAYPNKYEIYRRVDEPNGQERYVLLEEFVEKPTPDEITNAFRPRKNENEKSASG
FWGFLSGIL

>Os08t0118900

MAGVLRLAGAARSPLARALAPAARRMGASAAAAMEDEAYWTEWEEEEEKARARESAP
VAEMCPTGGGGGGPQWVVMGRPGPQKHAHAARLAEVLAVPYISMGTLVRQELSPASSLY
KKIANSVNEGKLVPEDIIFGLLTKRLEEGYNKGETGFILDGIPRTHMQAEILDEIVDIDLVLN
FKCADNCFMKRRFGGDICPHCGQLFDFSKTASSDRNPSLGSCTWPSQVQHAAVLGLEDSR
MEKMRAYAEQTKLLEDYYRKQRKLMELKTSARPGETWQGLVAALHLQHLDASPTPHKL
TM

>Os08t0109300

MASSMAATATLSPPVLSAERPTVRGGLFLPPSPATSRSLRLQSARRCGISPATRKPRSLPRAA
KVVVAVKADPLKVMIAGAPASGKGTQCELIKSKYGLVHISAGDLLRAEIAAGSENGKRAK
EFMEKGQLVPDEIVVNMVKERLLQPDAQEKGWLLDGYPRSYSQAMALETLNIRPDIFILL
DVPDELLVERVVGRRLDPVTGKIYHLKYSPPENEEIASRLTQRFDDTEEKVKLRLQTHYQN
VESLLSIYEDVIVEVKGDALVDDVFAEIDKQLTSSLDKKTEMVASA

>Os07t0412400

MASRGGGARRTRPNVLVTGTPGTGKTTTCSLLADAVDLRHINIGDLVREKSLHDGWDEEL
ECHIINEDLVCDELEDVMEEGGILVDYHGCDFFPERWFDLVVVLQTDNSILHDRLTSRGYM
GAKLTNNIECEIFQMLLEEARESYKEEIVMPLRSDNVEDISRNVGTLTEWINNWRPSRS

>Os03t0130400

MAAVQRLLRASASGGAAAAAAAARRRMSTAVAPEQTPAAAAFPFAAAAGRARQRVAEER
NVQWVFLGCPGVGKGTYASRLSRLLGVPHIATGDLVRDELASSGPLSVQLAEIVNQGKLV
SDEIIINLLSKRLKKGEEQGESGFILDGFPRTVKQAEILDGVTDIDMVVNLKLREDVLVEKC

LGRRICGQCGKNFNLACIDVKGENGLPPIYMAPLLPPNNCMSKLITRADDTEEVVRNRLQI
YNDMSQPVEGFYRQQGKLLEFDLPGGIPESWPKLLHVLNLEDQEEMKLATA

Potato (*Solanum tuberosum*)
>Sotub04g013920
MVVWTRAVVRTWRCRPTNFSRAFSEKLPTPEAKGRNVQWVFLGCPGVGKGTYAARLSK
LLGVPHIATGDLVRQQLSSHGPLALKLVDIVSQGQLISDEIVIDLLSKRLEAGEAKGEIGFIL
DGFPRTIRQAEILEGVTDIDLVINLKLREDALIAKCLGRRTCSECGGNYNVACIDMKGDDG
ETRMYMPPLLPPPHCETKLITRSDDTENVVKERLRIYHETSKPVEDFYRKRGKLLEFDLPG
GIPESWQKLLQALNIYDDDEDKKSAAA
>Sotub09g006620
MAAMIRLFRSSSSSSSNSISLISRSLSTAAASETVKSQSYPHNPHSTSVDPKAKTVQWVFLG
CPGVGKGTYASRLSTLLGVPHIATGDLVRDELKSSGPLSKQLAEIVNQGKLVSDEIILNLLS
KRLESGEAKGEAGFILDGFPRTVRQAEILTEVTDIDLVVNLKLPERVLIEKCLGRRICSECG
KNFNVASIDVAGENGAPRISMAPLNPPSQCVSKLITRADDTEAIVKERLSIYWDKSQPVED
FYRSQGKLLEFDLPGGIPESWPKLLEVLNLDEQEYKLSAAA
>Sotub03g005270
MALLSRIRAAVQPLIRTESLRSYGSAAAQLVDYDYDDYEYEEYQNRSYVMEESEGSIPRR
GVQWVIMGDPMAQRHVYAQWLSKLLDVPHISMGSLVRQELHPRSSLYKQIADAVNQGKL
VPEEVIFGLLSKRLEEGYCSGESGFILDGIPRSKIQAEILDKTVDIDLVLNLKCAEDLVSKKD
KSTGLYPPLEFLRRGASGISTSRQPEGGHFRPSSIMDDVSRKNLHVHAEQVNPLEEYYRKQ
RKLLDFQVAGGPGETWQGLLAALHLQHRNAVGSTQLTAGC
>Sotub02g037180
MAMLSFLGVSARPFLLAASSKSVRAYGSAAAAHFDYDYEEEPSGSVPRRGVQWLIMGDP
MTQRHVYAQWLSKLVDVPYISMGSLVRQELNPHYNKISSVVNEGKLVPEEVIFDLLSKRL
EEGYCRGENGFILDGIPRTMFQAIKRLEEYYRKQKKLLNYQVAGGPAETWRGLLAALQLQ
HMMSAVGSTQLTAGC
>Sotub03g023880
MSTSSVNLEDVPSESLMSELLRRMRCSSKPDKRLILIGPPGSGKGTQSPIIKDEYCLCHLAT
GDMLRAAVAAKTPLGIKAKEAMDKGELVSDDLVVGIIDEALKKPSCQKGFILDGFPRTVV
QAEKLDVMLQSRGTKVDKVLNFAIDDAILEERITGRWIHPASGRSYHTKFAPPKAPGIDDV
TGEPLIQRKDDTAAVLKSRLEAFHRQTEPVIDYYAKKGNVVNLPAEKPPQAVTAEVKKVL
S
>Sotub05g016010
MAASLEDVPSESLMSEVLRRLRCSSKPDKRLILIGPPGSGKGTQSPIIKDEYCLCHLATGDM
LRAAVAAKTPLGIKAKESMNNGELVSDDLVVGIIDEAMKKTSCQKGFILDGFPRTVVQAE
KLDEMLQKQGAKIDKVLNFAIDDAILEERITGRWIHPSSGRSYHTKFQPPKVPGVDDVTGE
PLIQRKDDTAEVLKSRLDAFHRQTEPVINYYSTKGVVASLHAEKPPKEVTSEVKNVLSS
>Sotub01g028550
MGTVVESANQGAVSLPTNKKVTVIFVLDLFPLLTWTGGPGSGKGTQCTNIVEHFGYTHLS
AGDLLRAEIKSGSENGTMISNMIKEGKIVPSEVTIKLLQRAIQENGNDKFLIDGFPRNEENR
AAFELVTGIEPEFVLFFDCPEAEMEKRLLGRNQGREDDNIETIRKRFNVYMESSLPVIEYY
NSKGKVRKIDAVKPVGEVFEAVKAVFTPANEKVKY
>Sotub08g022760

MDLHKEGDRGSAKQKKVKIVFVIGGPGSGKGTQCKRIAQQFGYTHLSVGEILRQEISSGS
ETGSMIQKIMKEGKLVPSDVTVRLLQQAMQGINSDKFLIDGFPRNEENVKAFEDLTKMEP
EFVLYLDCPQDEMEKRLLSRNEGREDDNIETIRKRFKVFMESTLPTIEYYESKGKIRKVDA
GKSVDEVFESIKVIFSQGKDNKVPPSRHKCKCLIL
>Sotub11g015570
MASCSSLNFTAVSSNPQKLPSSSISSPIVQRPFTSHLSFSKSSSLHSDQIPIRTHCGKLPQPNG
AGFVVLGCARKKEPLRIMISGAPASGKGTQCELITQKYGLVHIAAGDLLRAEIAAGSENGK
QAKEYMDKGKLVPNEIVVTMVKERLNGPDSREKGWLLDGYPRSSSQAIALEEFGFQPDL
FILLEVPEEILVERVVGRRLDPITGKIYHLKYSPPETEEIASRVTQRFDDTEEKACIVKLRLQ
THHQNVEAILLMYEDITVKVNGIGSKQEVFAQIDGALTQLLEQKQEKLGTVAA
>Sotub06g024300
MASCCSLSFSTVSSKPNKPYSSPISSSLQLPFTSQLPFSKKKSLYSNHTLLQTQCRKTPSPDC
PSFLVVGSAKKQEPLRVMISGAPASGKGTQCELITKKYDLVHIAAGDLLRAEIAAGTENGR
RAKEYMDKGQLVPNEIVVTMVKERLMRPDSQEKGWLLDGYPRSSSQAVALKEFQPDLFI
LLEVPEEILVERVVGRRLDPVTGRIYHLKYSLPETDEIAARLTQRFDDTEEKVKLRLHTHH
QNVESVLSMYKDTIFQVDGSVSKEEVFAQIDAALTQLLEAKE
>Sotub03g020180
MWRRFTSLPLFFSHLQQVRRADELKICQAFCTEIVKPPVEGESNSRRNIPFVAFVLGGPGSG
KGTQCLKIAETFGFDHIGAGDLLRKEIHSDSENGAMIQKLMKEGSIAPSEVTVKLIKKAIES
AENRKFLIDGFPRSEENRVAYERIIGAEPNFVLFFDCPEEVMVKRVLNRNEGRVDDNEHTV
KERLKVYKAITLPVANHYAKKGKLYKVDGTGTQEEIFERVRPIFASLRLST
>Sotub12g007490
MDVYRIGTLMELIRVLALSFADDGKRVKVCVQGSMGEGALAGMPLQLAGSRKILEYMD
WGDYGALGNFVNIGTIGGKEVEKQDDLFILVAPQNAVGNCIIDDMRAMTDAAGNRPIILV
NPKLKDLPASSGIMQTMGRDKRLEYAASFEICYQFRLLYYAGTQYPIMGALRMSYPYPYE
LYKRVDESPGKEKYISLATFAKRPSIDEMNDAFDGKSRNQEKKAEGFWGFLSGIL

Tomato (*Solanum lycopersicum*)
>SlADK1
MGTVVESANQGAVSLPTNKKVTVIFVLGGPGSGKGTQCANIVEHFGYTHLSAGDLLRAEI
KSGSENGTMISNMIKEGKIVPSEVTVKLLQRAIQENGNDKFLIDGFPRNEENRAAFELVTGI
EPEFVLFFDCPEAEMEKRLLGRNQGREDDNIETIKKRFNVYMESSLPVIEHYNSKGKVRKI
DAVKPVGEVFEAVKAVFAPSNEKVAA
>SlADK2
MAMLSFLGVSARTFLRAASSKSVRAYGSAVAAHFDYDNEEDMEEPSGSVPRRGVQWLIM
GHPMTQRHVYAQWLSKLMDVPYISMGSLVPQQLNPHYNKISSVVNEGKHVPEEVIFGLLS
KRLEEGHCRGENGFILDGIPRTMLQAEILDKVVDIDLVLNLKCSVSKNDRSNGIYSTEDQL
LKRGNLMSSRVMDGGAWKEKQYDHDEQIKPLEEYYRKQKKLLNYQVAGGPAETWQGL
LAALQLQHMMSAVGSTQLTAGC
>SlADK3
MALLSRIRAAAKPLIRTESLRSYGSAAAQLVDYDYDDYEYEEFQNRSCVMEESEGSVPRR
GVQWVIMGDPMAQRHVYAQWLSKLLGVPHISMGSLVRQELHPRSSLYKQIADAVNQGKL
VPEEVIFGLLSKRLEEGYCSGESGFILDGIPRSKIQAEILDKTVDIDLVLNLKRAEDLVSKKD
KSTGLYPPLEFLRMGASGISTSRQPEGGHFRPSSIMEDVSRKNLHVHAEQVNPLEEYYRKQ

RKLLDFQVAGGPGETWQGLLAALHLQHRNAVGSTQLTAGC
>SlADK4
MWRRRFTSLPLFFSHLQQVRRADELKICQAFCTETVKPPVEGESNSGRNSPFVAFVLGGPG
SGKGTQCLKIAETFGFDHIGAGDLLRKEMHSDSENGAMIQKLMKEGSIAPSEVTVKLIKK
AIESAENRKFLIDGFPRSEENRVAYERIIGAEPNFVLFFDCPEEVMVKRVLNRNEGRVDDNE
HTVKERLKVYKAITLPVANHYAMKGKLYKVDGTGTQEEIFERVRPIFASLRLST
>SlADK5
MSTSSVNLEDVPSESLMSELLRRMRCSSKPDKRLILIGPPGSGKGTQSPIIKDEYCLCHLAT
GDMLRAAVAAKTPLGIKAKEAMDKGELVSDDLVVGIIDEALKKPSCQKGFILDGFPRTVV
QAEKLDVMLQNRGTKVDKVLNFAIDDAILEERITGRWIHPASGRSYHTKFAPPKVPGIDDV
TGEPLIQRKDDTAAVLKSRLEAFHRQTEPVIDYYAKKGNVVNLPAEKPPQAVTAEVKKVL
S
>SlADK6
MVVWTRAVVRTWRCRPTNFSRAFSEKLPTSEPKGRNIQWVFLGCPGVGKGTYAARLSKL
LGVPHIATGDLVRQQLSSHGPLASKLVDIVSQGQLISDEIVIDLLSKRLEAGEAKGETGFILD
GFPRTIRQAEILEGVTDIDLVINLKLREDALIAKCLGRRTCSECGGNYNVACIDMKGDDGE
TRMYMPPLLPPPHCETKLITRSDDTENVVKERLRIYHEMSKPVEDFYRQRGKLLEFDLPG
GIPESWSKLLQALNIYDDEDKKSAAA
>SlADK7
MAASLEDVPSESLMSEVLRRLRCSSKPDKRLILIGPPGSGKGTQSPIIKDEYCLCHLATGDM
LRAAVAAKTPLGIKAKEAMNNGELVSDDLVVGIIDEAMKKPSCQKGFILDGFPRTVVQAE
KLDEMLQKQGSKIDKVLNFAIDDAILEERITGRWIHPSSGRSYHTKFQPPKVPGVDDVTGE
PLIQRKDDTAEVLKSRLDAFHRQTEPVINYYSTKGVVASLHAEKPPKEVTSEVKHVLSS
>SlADK8
MASCCSLSFSTVSSKPNKPYSSPISSSLELPFTSQLPFSKKYSLYSNHTLLQTQCRKTQSPDC
PSFLVVGSAKKQEPLRVMISGAPASGKGTQCELITKKYDLVHIAAGDLLRAEIAAGTENGR
RAKEYMDKGQLVPNEIVVTMVKERLMCPDSQEKGWLLDGYPRSLSQAVALKEFQPNLFI
LLEVPEEILVERVVGRRLDPVTGRIYHLKYSPPETDEIAARLTQRFDDTEEKVKLRLHTHR
QNVESVLSMYKDTIFQVDGSVSKEEVFAQIDGALTQLLEAKE
>SlADK9
MDLHKEGDTGSAKQKKVKIVFVIGGPGSGKGTQCKRIAQQFGYTHLSVGEILRQETSSGS
ETGHMVQKIMKEGKLVPSDVTVRLLQQAMQGIDNDKFLIDGFPRDEENVKAFEDLTKME
PEFVLYLDCPQDEMEKRLLSRNEGREDDNIETIRKRLKVFVESTLPTIEYYESKGKIRKVD
AGKSIDEVFESIKVIFSPGKDNKMPPSKHKCKCLIL
>SlADK10
MAAMIRLFRSSSSSSSLISRSLSTAAASETVKSRSYPHSTSVEPKAKSVQWVFLGCPGVGK
GTYASRLSTLLGVPHIATGDLVRDELKSSGPLSKQLAEIVNQGKLVSDEIILNLLSKRLESGE
AKGEAGFILDGFPRTVRQAEILTEVTDIDLVVNLKLPERVLVEKCLGRRICSECGKNFNVAS
IDVAGENGAPRISMAPLNPPSQCISKLITRADDTEAIVKERLSIYWDKSQPVEDFYRSQGKL
LEFDLPGGIPESWPKLLEVLNLDEQEHKLSAAA
>SlADK11
MAMIASVTMNFPHISTHNISSNQTFSPICTNNPSNFSSSSSSSIPISSNSIRLSSSIAYSEQLIAS
HNVNRRTKNRKIKVISARSEPLKVMISGAPASEKDVVGWMKSLYPLQEVSGLSNGSGEIS
GSERFLLQYWSYATQILFSQTNGLIEQQKFVFCGFGLVHISTGDLLRAELSAGTDIGNKAKE

YMNAGRLVPDEIVTAMVTTRLSKEDAKEKGWLLDGYPRTLAQAESLERLNIRPDIYIVLD
VPDAILIDRCVGRRLDPLTGKIYHVTNFPPETEDIKARLITRPDDTEEKVKSRLQIYKQNAE
AILPVYSDIMNKIDGNRGKDSVFAEIDSLLSRVQKEEQDARKSEESAISSTRADMASLSKD
WRGIPTRLNNIPHSREIREYFYTDVLQATQRAVNDGKTRLKIEINIPELNPSMDVYRIGTLM
ELIRVLALSFADDGKRVKVCVQGSMGEGALAGMPLQLAGSRKILEYMDWGDYGALGNFI
NIGSIGGKEVEKQDDVFILVAPQNAVGNCIIDDMRAMTDAAGNRPIILVNPKLKDLPASSGI
MQTMGRDKRLEYAALFEICYQFRLLYYAGTQYPIMGALRMSYPYPYELYKRVDESPGKE
KYISLATFAKRPSIDEMNDAFEGKSRNQEKKAEGFWYVINYPPFYLCFE
